# Composite MAX phase/MXene/Ni electrodes with a porous 3D structure for hydrogen evolution and energy storage application†

**DOI:** 10.1039/d3ra07335a

**Published:** 2024-01-18

**Authors:** Sergii A. Sergiienko, Luc Lajaunie, Enrique Rodríguez-Castellón, Gabriel Constantinescu, Daniela V. Lopes, Nataliya D. Shcherban, José J. Calvino, João A. Labrincha, Zdenek Sofer, Andrei V. Kovalevsky

**Affiliations:** a Department of Inorganic Chemistry, University of Chemistry and Technology Prague Technická 5, 166 28 Prague 6 Czech Republic sergiiee@vscht.cz sergeenko_sergei@ukr.net; b Department of Materials and Ceramics Engineering, CICECO – Aveiro Institute of Materials, University of Aveiro 3810-193 Aveiro Portugal akavaleuski@ua.pt; c Departamento de Ciencia de Los Materiales e Ingeniería Metalúrgica y Química Inorgánica, Facultad de Ciencias, Universidad de Cádiz Campus Río San Pedro S/N, Puerto Real 11510 Cádiz Spain; d Instituto Universitario de Investigación de Microscopía Electrónica y Materiales (IMEYMAT), Facultad de Ciencias, Universidad de Cádiz Campus Río San Pedro S/N, Puerto Real 11510 Cádiz Spain; e Department of Inorganic Chemistry, Faculty of Sciences, University of Malaga 29071 Malaga Spain; f L. V. Pisarzhevsky Institute of Physical Chemistry of NAS of Ukraine 31 Nauki Ave. Kyiv 03028 Ukraine

## Abstract

MXenes, a family of two-dimensional (2D) transition metal carbides, have been discovered as exciting candidates for various energy storage and conversion applications, including green hydrogen production by water splitting. Today, these materials mostly remain interesting objects for in-depth fundamental studies and scientific curiosity due to issues related to their preparation and environmental stability, limiting potential industrial applications. This work proposes a simple and inexpensive concept of composite electrodes composed of molybdenum- and titanium-containing MAX phases and MXene as functional materials. The concept is based on the modification of the initial MAX phase by the addition of metallic Ni, tuning Al- and carbon content and synthesis conditions, followed by fluoride-free etching under alkaline conditions. The proposed methodology allows producing a composite electrode with a well-developed 3D porous MAX phase-based structure acting as a support for electrocatalytic species, including MXene, and possessing good mechanical integrity. Electrochemical tests have shown a high electrochemical activity of such electrodes towards the hydrogen evolution reaction (HER), combined with a relatively high areal capacitance (up to 10 F cm^−2^).

## Abstract 2

The MAX phase/MXene/Ni composite with 3D porous structure prepared was assessed for energy conversion and storage application, using the hydrogen evolution reaction under alkaline conditions as a model system.

## Graphical abstract

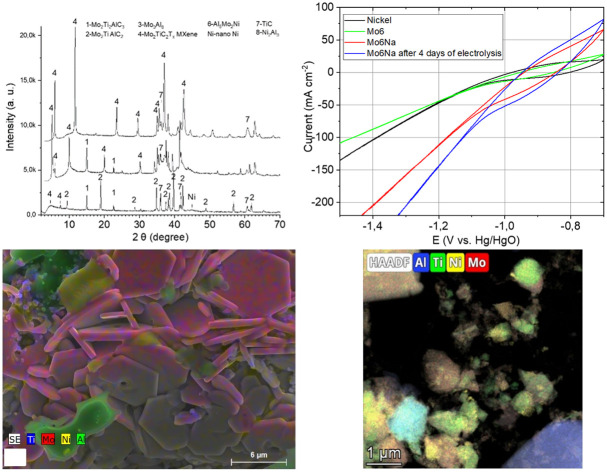

## 1. Introduction

Nowadays, the exploitation of renewable wind and solar energy for sustainable development is an important part of national and international energy policies. However, due to the variability of wind and solar power, energy storage devices are required as enabling technology for renewables integration, improving grid reliability and allowing effective management of power generation.^1^ Electrochemical energy storage systems, such as metal-ion batteries and supercapacitors (SCs), are considered promising ways to store renewable energy. However, their performance hardly meets the growing demand for large-scale energy storage. To solve this issue, new materials with better performance are required for energy storage systems.^2^ Large-scale renewable energy storage technologies have grown rapidly in the last few years. The production of hydrogen by water electrolysis is now considered one of the most promising technologies. Also, ammonia is a potential platform for hydrogen storage; while the NH_3_ electrosynthesis from N_2_ and H_2_O looks attractive, it is currently limited by its low efficiency.^3^ Another attractive option for energy storage in the form of renewable energy is the production of liquid fuels by electrochemical CO_2_ reduction.^4^ All these technologies require efficient and cheap electrocatalysts for successful industrial implementation.

Alkaline water electrolysis is an industrially well-established technology enabling efficient energy conversion and storage.^5^ Among various electrocatalysts for HER in alkaline medium,^6,7^ there are several that meet the requirements of low cost, sufficiently high catalytic activity and stability, including transition metal carbides and transition metal-based alloys. Transition metal carbides exhibit catalytic activity close to that of Pt, have low cost and high abundance, but have a tendency to oxidize and lower electrical conductivity compared to metals.^8^ Among the transition metal-based alloys, Ni–Mo alloys are the most active and stable HER electrocatalysts with low overpotential. Their high electrochemical activity is attributed to the synergistic effects provided by adjacent atoms of Ni and Mo, resulting in unsaturated d-orbitals, similar to Pt.^9^ However, in economical water-alkali electrolysers, the sluggish water dissociation kinetics (Volmer step) in platinum-free electrocatalysts results in poor hydrogen-production activity.

MXenes, a family of two-dimensional (2D) transition metal carbides, attract attention as electrodes for HER due to the combination of a large electrochemically active surface, high conductivity and hydrophilic nature provided by their hydroxyl- or oxygen-terminated surfaces.^10^ The water adsorption on MXenes is exothermic and facilitates its dissociation (Volmer step).^11^ All these properties make MXenes promising as electrocatalysts for several processes: HER in an alkaline medium, where water dissociation is a rate-limiting step,^5^ as well as for the nitrogen reduction reaction (NRR),^3^ and the CO_2_ reduction reaction (CO_2_RR).^4^ In fact, even the MAX phases themselves can be used as electrocatalysts, among which Mo_2_TiAlC_2_ was identified as the most catalytically active.^12^ Therefore, the combination of 2D transition metal carbides (MXenes) with transition metal-based alloys (Ni and Mo) is potentially a very promising way to achieve high electrocatalytic performance. The unique properties of MXenes also make them a promising electrode material for supercapacitors (SC)^13^ and metal ion batteries (MIBs).^14^

Several issues limit the widespread industrialization of MXenes. Most of the synthesis methods are complicated and require a preliminary MAX phase grinding, before etching and delamination. This destroys the three-dimensional (3D) MAX phase structure.^15,16^ However, it is critically important to keep the intrinsic layered nanostructure at bulk scale, with those two outstanding properties together: high specific surface area and high electrical conductivity.^17^

Another issue is related to the designing of an efficient electrode structure, making full use of active materials and facilitating the transport of ions and electrons to the active sites. Porous architectures can serve as ion-buffering reservoirs to enhance electrolyte ions delivery and reduce the tortuosity of electrode materials so that ions can travel directly through channels.^18^ For example, nanoporous metal electrodes in supercapacitors and batteries should have large pores (50–100 nm) for rapid ion diffusion and small pores to ensure a large internal surface area for charge storage.^19^

Another important parameter for bulk MXene-based electrodes is electrical conductivity. In general, it can be seen that thinner MXene-based electrodes exhibit higher specific capacitance due to lower ion transport resistance compared to thick electrodes.^13,20^ Thick electrodes usually have higher electrical resistance due to a greater number of inter-flake interfaces, similar to the known difference between the resistivities of a single monolayer Ti_3_C_2_T_*x*_ flake and a multi-layered film of around one order of magnitude higher.^21^ This is particularly important when the MXene flake size decreases, and the inter-flake resistance increases.^22^ At the same time, when the electrode thickness decreases, the areal capacitance and the total electrocatalytic activity usually decrease, limiting the potential practical applications.^23^ To achieve high values of areal capacitance and catalytic activity, an increase in the thickness of the active electrode material is required.

Thus, a simultaneous improvement of the conductivity of bulk multi-layered MXene based electrodes is desirable. This can be performed by several methods: (1) by the formation of nano-metal/MXene composites, where the nano-metal (Ni) will act as an internal current collector at the interflake interfaces, (2) by the formation of the MXene 3D structure without destroying the structure of the MAX phase and therefore, maintain the C–C bonds between the MXene crystals.

Similar to other 2D materials, MXene nanosheets tend to stack and aggregate due to strong van der Waals forces, greatly limiting their performance in practical applications. Compared to the 2D layered structure, a 3D porous MXene structure appears to be a feasible solution to tackle the restacking problem caused by van der Waals forces and hydrogen bonding between the 2D nanosheets.^24^ Taking into account the above-mentioned issues and the existing discussions in the literature,^25^ the development of three-dimensional (3D) nanoarchitectures using 2D MXene flakes is currently one of the most important research challenges, along with improving of the stability of MXenes.

Electrodes based on Ti_3_C_2_(OH)_*x*_, Mo_2_TiC_2_(OH)_*x*_, Mo_2_Ti_2_C_3_(OH)_*x*_ MXenes are among the most active HER electrocatalysts.^26–28^ Although some noble and non-noble metals with 3D porous structure have been fabricated by dealloying,^29^ the most interesting for practical application are nano-metal/MXene composites based on inexpensive metals (*e.g.* Ni, Mo, Ti) and possessing a high activity toward HER.

Therefore, this work intends to contribute to this progress by developing reliable and inexpensive electrode concepts for electrochemical energy conversion and storage systems, promoted by a facile method of nano-metal/MXene (metal – Ni, MXene – Mo_2_TiC_2_(OH)_*x*_) composites formation with 3D porous nanostructure by direct alkaline etching of metal–Al alloy and MAX phase composites.

## 2. Material and methods

### 2.1 Synthesis and preparation of materials

The samples were synthesized from commercially available powders of titanium (Alfa Aesar, 10386, −325 mesh, less than 45 microns, 99%), carbon black acetylene (Alfa Aesar, 39724, 50% compressed, 99.9+%), nickel (Alfa Aesar, 10256, APS 3–7 micron, 99.9%). Nanometric Al powder (99.995%, 770 nm metal basis, Nanografi, CAS number: 7429-90-5) was used as a source of Al. As an alternative, the micrometric Al powder (325 mesh, 99.5%, metal basis, APS 7–15 microns, Alfa Aesar, CAS number: 7429-90-5) was used for the synthesis of one sample (Mo6Al_micro_). Carbon black was subjected to ball milling for 8 hours (2 g of carbon black and 30 ml of ethanol) to decrease particle size. The starting powders were sequentially mixed, followed by ball milling for 2 hours with ethanol to obtain a suitable homogeneous mixture (6 g of powders mixture and 20 ml of ethanol). Next, the powder mixtures were pressed (10 kN) uniaxially in the shape of discs (diameter = 25 mm, thickness approx. 3 mm). The sintering of the powder compacts was performed under an Ar atmosphere at 1500 °C for 3 hours. The samples were placed on top of the carbon black layer in an alumina crucible and were also covered with a carbon black layer to reduce the evaporation of Al. Initial compositions, corresponding denominations and processing parameters of the samples are listed in Tables 1 and 2. To prepare powders from initial sintered products (MAX phases) for XRD analysis, the sintered products were ground to decrease the average particle size down to about 50 μm.

**Table 1:** Denominations, initial composition of the samples (electrodes) and etching time in 10 M NaOH solution

Samples (before etching)	Initial composition, Ni : Mo : Ti : Al : C, molar ratios					Etching time, days	Samples (after etching)
	Ni	Mo	Ti	Al	C		
Mo0		2	1	1	2	—	—
Mo1		2	1	**6**	2	60 d	Mo1Na
Mo2	1	2	1	**6**	2	60 d	Mo2Na
Mo3^a^		2	1	**8**	2	10 d	Mo3Na
Mo4^a^	1	2	1	**8**	2	10 d	Mo4Na
Mo5		2	1	7	**2**	60 d	Mo5Na
Mo6	1	2	1	7	**2**	60 d	Mo6Na
Mo6a	1	2	1	7	**2**	15 d	Mo6aNaT70
Mo6Al_micro_	1	2	1	7	**2**	15 d	Mo6Al_micro_NaT70
Mo7		2	1	7	**3**	30 d	Mo7Na
Mo8	1	2	1	7	**3**	30 d	Mo8Na
Mo9^a^		2	1	7	**4**	1 d	Mo9Na
Mo10^a^	1	2	1	7	**4**	1 d	Mo10Na
Mo11^a^		2	1	**10**	2	1 d	Mo11Na
Mo12^a^	1	2	1	**10**	2	1 d	Mo12Na

a Sample destroyed after etching.

**Table 2:** Parameters of several representative samples

Samples parameters	Sample Mo5	Sample Mo6
After pressing and before sintering		
Initial composition, Ni : Mo : Ti : Al : C, molar ratios	0 : 2 : 1 : 7 : 2	1 : 2 : 1 : 7 : 2
Weight, g	4	4
Geometrical dimensions (diameter, thickness), mm	25, 3.5	25, 3.5
After sintering for 3 h at 1500 °C		
Weight, g	3.85	3.93
Geometrical dimensions (diameter, thickness), mm	25.1, 5	22.8, 3.4
	Sample Mo5Na	Sample Mo6Na
After etching during 60 days		
Weight, g	2.06	2.57
Geometrical dimensions (diameter, thickness), mm	21, 3	22.8, 3.4

### 2.2 Preparation of electrodes

**Fig. 1:**
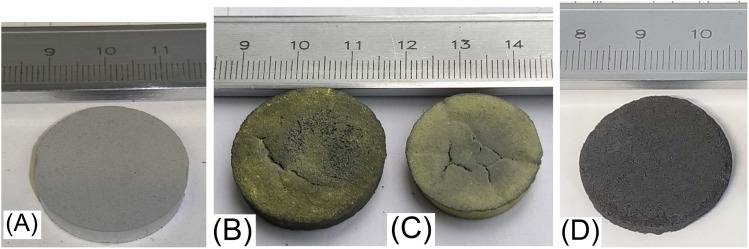
Photographic images of the samples: Mo6 after pressing (A), Mo5 after sintering (B), Mo6 after sintering (C), Mo6 after sintering and etching in 10 M NaOH (D).

The electrodes were produced directly by sintering initial (Ti, Mo, Al, C and Ni) pressed powders (Fig. 1A–C). The composite electrodes of MXene/MAX phase or MXene/MAX phase/nano-Ni were formed by etching the sintered products in excess of 10 M NaOH solution at room temperature and after moderate heating. The sample was placed in a 100 ml solution and kept for 10 days, then the solution was replaced with a new one, and etching continued with constant stirring of the solution. After the etching process, the samples were washed in distilled water. A representative image of the Mo6Na sample (after etching) is presented in Fig. 1D.

Also, for the sake of comparison, etching of Al and Ni to form MXene from the sintered and milled Mo6 sample was carried out in HF solution (2 g of powder was added to 40 ml of 50 wt% HF aqueous solution) at 60 °C for 3 and 6 days. As a next step, the obtained powders were washed several times with distilled water (at room temperature), filtered using vacuum filtration with a conventional filter paper and dried at room temperature.

### 2.3 Material characterization

The phase composition was assessed by X-ray diffraction analysis (XRD), using a Rigaku D/Max-B diffractometer system (Cu Kα radiation, 0.154056 nm).

High-resolution (scanning) transmission electron microscope imaging (HR-(S)TEM) was performed by using a FEI Talos microscope operated at 200 kV. The Talos was equipped with an X-FEG gun, a super EDS detector, several imaging detectors including high-angle annular dark-field imaging (HAADF), and a Gatan Energy Filter (Quantum ERS) which allows for working in dual-electron energy loss spectroscopy (EELS) mode. Automatic indexation of the selected area electron diffractograms (SAED) and Fast Fourier Transform (FFT) patterns was performed by using the JEMS software.

Hitachi SU-70 scanning electron microscopy (SEM) combined with energy dispersive spectroscopy (EDS) analysis (Bruker Quantax 400 detector) was used to characterize the relevant microstructural features.

X-Ray Photoelectron Spectroscopy (XPS) measurements were carried out using a Physical Electronics. VersaProbe II scanning XPS photoelectron spectrometer equipped with monochromatic X-ray Al Kα source (1486.6 eV). A charge neutralizer operating at a vacuum better than 10^−7^ Pa was used. The sample charging effects were excluded by charge reference against adventitious carbon (C 1s at 284.8 eV). An estimated error of ±0.1 eV was used for all analyses. Spectra were recorded in constant pass energy mode at 29.35 eV using a 100 μm diameter analysis area. The PHI ACCESS ESCA-V6.0 F software package and Multipak v8.2b were used for acquisition and data analysis, respectively. A Shirley-type background was subtracted from the signals. Recorded spectra were fitted using Gauss–Lorentz curves to determine the binding energy of the different element core levels more accurately. Samples were also studied after etching with an Ar^+^ gun (4 kV, 2 × 2 mm^2^) for 6 min.

### 2.4 Electrochemical measurements

Electrochemical measurements were carried out using an Autolab potentiostat (PGSTAT 302) connected to the electrochemical three-electrode cell. A Pt wire was used as a counter electrode (CE) and Hg|HgO|NaOH (1 M) (+0.098 V *versus* saturated hydrogen electrode) as a reference electrode connected by a Luggin capillary to the electrolyte as previously described in publication.^30^ All experiments were performed at room temperature with NaOH (1 M) as an electrolyte. Each working electrode tested had 25 mm in diameter, about 3 mm in thickness and was composed of ∼4 g of the material before etching. Hydrogen evolution tests were performed between −0.7 and −1.5 V with a scanning rate of 10 mV s^−1^. The current density values were calculated from the geometric area of each electrode (∼10 cm^2^).

To estimate the electrocatalysts' surface area (ESCA), the capacitance method was chosen.^31^ The ECSA can be calculated from the double-layer capacitance (*C*_dl_) as: ECSA = *C*_dl_/*C*_s_. The specific capacitance values (*C*_s_) for a flat standard with 1 cm^2^ of the real surface area that is generally in the range of 20 to 60 μF cm^−2^ (40 μF cm^−2^ was taken as the average value).^26^ To estimate *C*_dl_, cyclic voltammetry (CV) can be used, and these data were recorded around the open circuit potential (OCP) using different scan rates (*V*_b_) from 1 to 120 mV s^−1^. Further, the double-layer capacitance can be calculated as *C*_dl_ = Δ*J*/*V*_b_. The values of double-layer capacitance (*C*_dl_) for samples can be estimated by plotting the Δ*J* = (*J*_a_ − *J*_c_)/2 (at overpotential in the middle of the cycling range) against the CV scan rate, *J*_a_ and *J*_c_ are the anodic and cathodic current densities, respectively. Also, the *C*_dl_ value can be estimated using electrochemical impedance spectroscopy (EIS) from the Nyquist plot by fitting data using the equivalent circuit.^32^ The ECSA values were represented per unit of surface area (areal capacitances, F cm^−2^), and mass (gravimetric capacitances, F g^−1^) of the samples. This seems to be a suitable approximation because the specific capacitance measured also includes the pseudocapacitance in addition to electrical double-layer capacitance.^33,34^

The values of areal capacitance (*C*_3_) were estimated by two methods, first from CV curves and second using electrochemical impedance spectroscopy (EIS) data.

The values of areal capacitance (*C*_3_) were calculated from CV curves recorded in the range from −0.7 to −1 V using the simplified formula *C* = Δ*J*/*V*_b_, and the values of Δ*J* were obtained around the open circuit potential (OCP) approximately at −0.85 V, at a scan rate *V*_b_ of 1 mV s^−1^. As discussed below, the conventional method of capacitance calculation by current integration over time^35^ may not be suitable in this case.

The electrochemical impedance spectroscopy (EIS) was carried out using the same potentiostat at open circuit potential, with a 50 mV of signal amplitude and frequencies ranging from 20 kHz to 5 mHz.^36^

## 3. Results and discussions

As reported earlier, a modified Ti_3_C_2_Al_*z*_ (*z* > 1) MAX phase was synthesized.^37,38^ The bulk structure of such modified Ti_3_C_2_Al_*z*_ (*z* > 1) MAX phase probably consists of crystals of the conventional Ti_3_AlC_2_ and modified Ti_3_Al_2_C_2_ MAX phases, similar to the previously synthesized Mo_2_Ga_2_C MAX phase.^39^ Aluminium excess facilitates etching and the formation of Ti_3_C_2_(OH)_*x*_ MXene layer even under alkaline conditions using 10 M NaOH. Still, the electrochemical performance of such MXene-containing electrodes is limited by the high electrolyte ion-diffusion resistance. Similar to porous graphene materials with 3D architecture and possessing enhanced ion transport kinetics,^40^ such MXene-based structures can be formed by assembling 2D MXene nanosheets obtained by conventional MAX phase etching using fluorine media.^24,41–43^ The MAX phase structure (crystal shape and size, porous structure) can be modified by changing the sintering temperature and time and also by the tuning initial composition (Al content), and by the formation of metal–Al/MAX phase composites.^37^ Al in reaction mixture acts as an essential component of the MAX phase. An additional contribution is to provide the conditions where Al melt plays the role of “solvent” and medium in which the modified MAX phase crystals grow with a chaotic crystal orientation.^37^ Therefore, it can be assumed that by selecting the sintering temperature, time and composition, it is possible to form the MAX phase with the desired crystal size and porosity suitable for the transformation into well-performing MXene-containing electrode.

Therefore, our strategy to develop an MXene-containing electrode with 3D porous structure is based on the modification of the initial MAX phase by tuning the crystal structure, the nominal composition and the subsequent selection of the etching conditions.

### 3.1 Preparation of Mo- and Ti-based MAX phases/MXenes composites: the influence of Al and C content

Usually, high temperature and long annealing time and pressure are required for the synthesis of the Mo_2_TiC_2_Al MAX phase (4 hours at 1600 °C).^44^ Our results have shown that the Al excess in the reaction mixture can promote the MAX phase (Mo_2_Ti_2_AlC_3_, Mo_2_TiAlC_2_) formation process at a lower temperature, probably generating Al-rich metal–Al alloys (intermediates) with a lower melting point (*e.g.* MoAl_4_ with *T*_melt_ = 1130 °C, and TiAl_3_ with *T*_melt_ = 1340 °C^45^). The presence of such alloys is expected to accelerate the diffusion of the components and improve the mass transfer mechanisms in the reaction mixture. In the literature, such a process is commonly called liquid phase sintering (LPS).^46^ In the case of the obtained samples (Table 1), sintering at 1500 °C for 3 hours (lower than the melting point of Mo_3_Al_8_, *T*_melt_ = 1570 °C^47^) allowed to form composites with a high MAX phase content attained for a wide range of Al content in the initial mixture (Mo : Ti : C : Al = 2 : 1 : 2 : *x*, from *x* = 1 to 8).

**Fig. 2:**
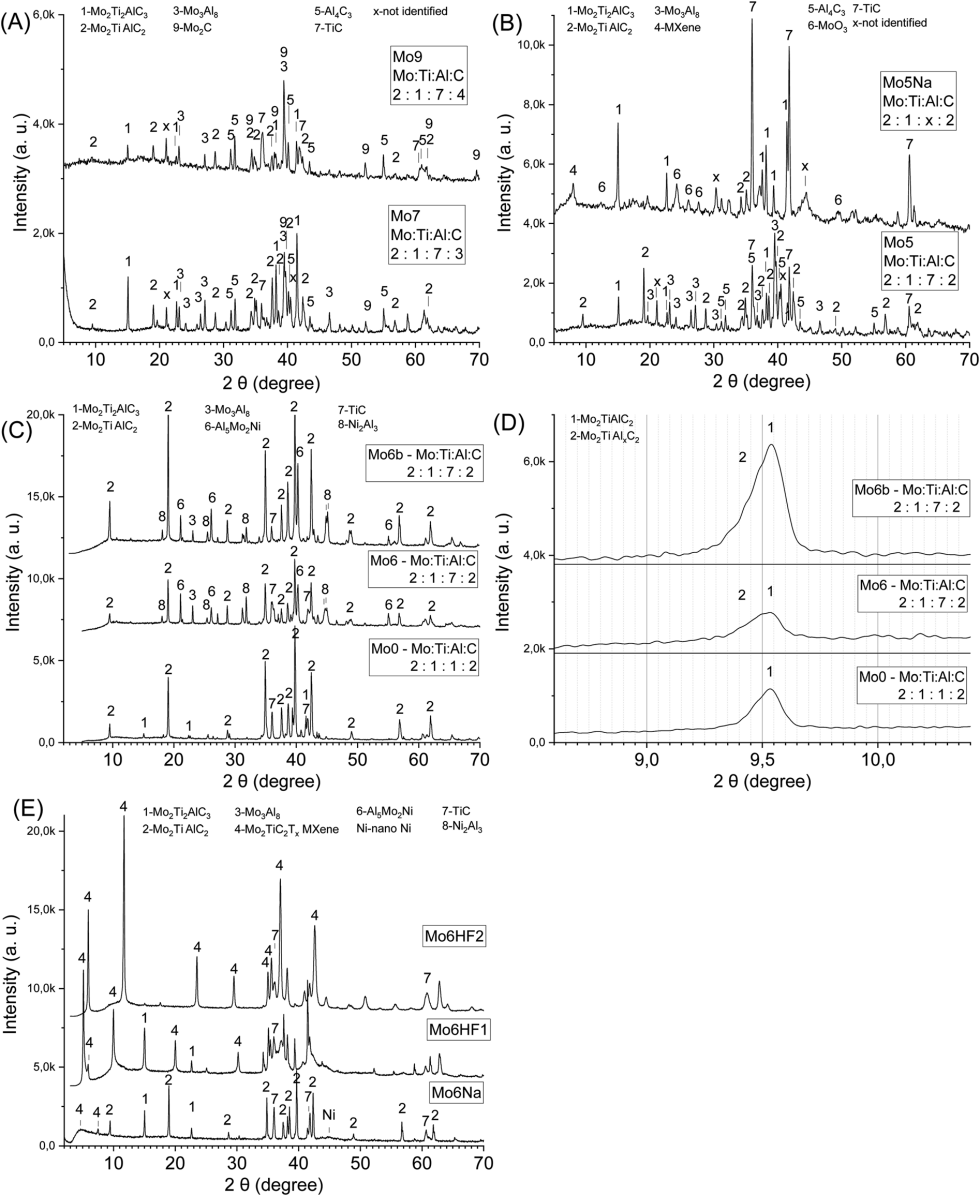
The influence of carbon content combined with an Al excess on the phase composition: the samples Mo5, 7, 9 (A); the sample Mo5 after sintering (before etching), and Mo5Na after etching in 10 M NaOH during 60 days (B); the influence of aluminium, nickel content and sintering temperature on the phase composition: the samples Mo0 – Mo : Ti : Al : C = 2 : 1 : 1 : 2 sintered at 1600 °C, Mo6 – Ni : Mo : Ti : Al : C = 1 : 2 : 1 : 7 : 2 sintered at 1500 °C, Mo6b – Ni : Mo : Ti : Al : C = 1 : 2 : 1 : 7 : 2 sintered at 1600 °C (C); small-angle X-ray diffraction patterns for samples from (C) (D); Mo6Na after etching in 10 M NaOH 60 days, Mo6HF1 after etching in 50% HF 3 days, Mo6HF2 after etching in 50% HF 6 days (E).

As a complementary approach, varying the carbon content with high Al content (Mo : Ti : C : Al = 2 : 1 : *k* : 7, *k* = 2, 3, 4) allows tuning the Mo_2_TiC_2_Al and Mo_2_Ti_2_C_3_Al MAX phases ratio in the sintered samples Mo5, Mo7, Mo9 (Table 1, Fig. 2A and B). The increase in carbon content (from *k* = 2 to 3) in the initial reaction mixture promotes the formation of carbon-rich MAX phase (Mo_2_Ti_2_C_3_Al), further addition of carbon (*k* = 4) increases the content of conventional carbides (TiC, Mo_2_C). Other phases (Mo_3_Al_8_, Al_4_C_3_) are also present in the sintered products. Due to the high brittleness of samples with high carbon content (*k* = 3, 4) and the presence of a significant amount of conventional carbides, further experiments were carried out with the samples that are expected to have better electrical properties (*k* = 2), combined with suitable mechanical properties allowing electrochemical testing.

The small angle diffraction peak (2*θ* ≈ 9.5°) in XRD patterns of the obtained samples (Mo5, Mo7, Mo9, Table 1, Fig. 2A and B) related to *c*-lattice parameter of Mo_2_TiAlC_2_ MAX phase do not represent peaks shift or broadening compared to the Ti_3_AlC_2_ MAX phase where more than one Al layer was introduced between the Ti_3_C_2_ layers.^37^ Therefore, the incorporation of additional Al likely takes place between the multilayered MAX phase crystals (Mo_2_TiAlC_2_ or Mo_2_Ti_2_C_3_Al) and not between the individual layers. It can be assumed that the driving force for the inclusion process of A atoms between the MX layers in the MAX phases is associated with M–A bond binding energy. Hence, if the M–A bond energy is strong enough than A–A, and there is an excess of A element in the reaction mixture, then the formation of modified MAX phase containing more than one layer of A element might be favourable. The M–A bonds in the MAX phases and in Ti_3_C_2_Al, in particular, have a weak covalent character,^48^ with the Al 2p binding energy equal to 72.1 eV; for pure Al this value is equal to 72.8 eV.^49^ For Mo_2_TiAlC_2_, the Al 2p binding energy (73.4 eV^50^) is higher than for elemental Al, although the difference between these values is small. These values of binding energy correspond to ambient conditions, but, as is well-known, the binding energy (enthalpy) is a temperature-dependent parameter,^51^ and is directly related to the melting temperature of solids.^52,53^ For Al *T*_melt_ = 660 °C, and most MAX phases are relatively stable up to 1300 °C in vacuum, then decomposition occurs due to evaporation of Al.^54^ Thus, it can be assumed that at high temperature the binding energy of A element in MAX phase (*e.g.* Al in Ti_3_C_2_Al) might be higher than in pure A element (*e.g.* Al) melt; this could facilitate the formation of conventional MAX phases with one layer of A element and modified MAX phases with more than one layer of A element. Other factors should also be taken into account in the case of Mo_2_TiAlC_2_ or Mo_2_Ti_2_C_3_Al phases. Most of the additional Al in the obtained samples is incorporated into solid Mo_3_Al_8_ alloy (Fig. 2A and B) having a melting temperature *T*_melt_ = 1570 °C that was applied during the synthesis (1500 °C). This likely restricts the penetration of extra Al atoms between the individual MAX phase layers. In the synthesis of the modified Ti_3_C_2_Al_*z*_ (*z* > 1) MAX phase,^37^ the SHS method was involved. This method is characterized by a short reaction time and high temperature exceeding 1500 °C. Under these conditions, TiAl_3_, which has a melting point *T*_melt_ = 1340 °C,^45^ also melts, so it does not restrict the formation of the modified Ti_3_C_2_Al_*z*_ (*z* > 1) MAX phase. The short reaction time during SHS allows to avoid considerable crystal growth of the MAX phase that possibly also facilitates the introduction of additional Al between the layers. Thus, the presence of a sufficient excess of free A element in the reaction mixture, the high temperature and the small crystal size of the MAX phase are likely to promote the formation of modified MAX phases with more than one layer of A element.

To form an MXene-based porous electrode structure, etching conditions were optimized by tuning temperature and time. Etching of such bulk sintered samples at elevated temperature (80–100 °C) in 10 M NaOH can intensify the process and reduce time, but, as the following studies reveal, the electrocatalytic activity of these samples towards HER was also slightly reduced, probably due to partial MXene oxidation and formation of carbo-oxides.^55^ Therefore, a further etching process was carried out at a temperature not higher than 70 °C optimizing the etching time, although this process is long.

Optimization of the Al amount in such composites plays an important role in the formation of the porous MAX phase/MXene structure after Al etching. To determine the optimal Al content, samples after sintering with different Al and C ratios were etched in 10 M NaOH at room temperature (Mo : Ti : C : Al = 2 : 1 : 2 : *x* and 2 : 1 : 3 : *x*, *x* was changed up to 8). Samples with high Al content (*x* = 8) were found to be destroyed during the etching process (10 days at 20 °C). With a lower Al content (*x* = 6), the etching process is slow, and the thickness of the etched layer reaches several tens of microns. The etching of samples with Al content *x* = 7 during 60 days at 20 °C allows obtaining a porous composite with a monolithic structure (Fig. 1D). At the same time, the thickness of the etched layer reaches the thickness of the sample (3.5 mm). After etching of such composites, the XRD peaks related to the several compounds (Mo_2_TiC_2_Al, Mo_3_Al_8_, Al_4_C_3_) disappear (Fig. 2B, samples Mo5, Mo5Na), and a small-angle peak in the range 7–8.5° related to MXene is observed (Fig. 2B, sample Mo5Na). This small angle peak likely belongs to the Mo_2_TiC_2_(OH)_*x*_ MXene, since the position of this peak is the same as for the non-delaminated HF-etched MXene described in the literature.^26,56^ Thus, the obtained results demonstrate the possibility of Mo_2_TiC_2_(OH)_*x*_ MXene synthesis by alkaline etching of the corresponding MAX phase. The formation of nanostructured molybdenum oxide (MoO_3_) was also observed (Fig. 2B, sample Mo5Na), in agreement with literature data suggesting partial oxidation and dissolution of Mo in the form of molybdate ion (MoO_4_^2−^) during etching of the Mo_3_Al_8_ phase in 1 M KOH solution.^57,58^ Therefore, the Al etching of Mo_2_TiC_2_Al and, possibly also partial Mo dissolution is responsible for the formation of free space between the Mo_2_TiC_2_(OH)_*x*_ MXene layers and the observed appearance of a small-angle peak in the range 7–8.5°. In addition, several new unidentified new peaks in the XRD pattern of the Mo5Na sample were observed at 30.3° and 44.4°.

Assuming that the thickness of a layer of Mo_2_TiC_2_(OH)_*x*_ MXene is approximately 1 nm^14^ and the calculated values of interlayer spacing *ca.* 1.26 nm (for 2*θ* ≈ 7°), 1.11 nm (for 2*θ* ≈ 8°) and 1.04 nm (for 2*θ* ≈ 8.5°), it is possible to estimate the pore size between the MXene layers as approximately in the range of 0.04–0.26 nm, which is close to the water molecule size (0.28 nm). Hence, a longer etching time or elevated temperature is required to facilitate further Al etching under the above conditions due to high diffusion limitations.

### 3.2 Preparation of Ni-containing Mo- and Ti-based MAX phases/MXenes composites

Processing of Ni-containing Mo- and Ti-based MAX phases/MXenes composites can be successfully performed by sintering the mixture of starting components at 1500 °C for 3 h. The resulting materials show a high content of Mo_2_TiC_2_Al and Mo_2_Ti_2_C_3_Al MAX phases formed within a wide range of Al concentration in the initial mixture Ni : Mo : Ti : C : Al = 1 : 2 : 1 : 2 : *x* (*x* = 1 to 8) (Fig. 2C and D). The small angle diffraction peak (2*θ* ≈ 9.5°) related to Mo_2_TiC_2_Al MAX phase in samples with Ni and high Al content (Mo6, Mo6b) became wider compared to the Mo_2_TiC_2_Al MAX phase prepared without Ni and with low Al content (Fig. 2C and D). The difference between the left (2*θ* ≈ 9.31, *d*-spacing ≈ 0.95 nm) and the right (2*θ* ≈ 9.64, *d*-spacing ≈ 0.91 nm) side of the peak at half-maximum is 0.04 nm. This indicates an expansion of the *c*-lattice parameter caused probably by the inclusion of additional Al between the layers and formation of modified Mo_2_TiC_2_Al_*x*_ MAX phase (*x* ≥ 1).^37^ The peaks in the diffraction patterns related to Mo_2_Ti_2_C_3_Al MAX phase become visible only after etching of such composite in 10 M NaOH or HF solution (Fig. 2E).

The rest of Mo, Ti, Al, C and Ni, which was not incorporated into the MAX phases of such composites, is present mainly in the form of TiC or Mo_3_Al_8_, Al_5_Mo_2_Ni and Ni_2_Al_3_ alloys filling the space between the MAX phase crystals. Perhaps this is the reason why the reaction products also contain Mo_2_Ti_2_C_3_Al MAX phase with a lower content of molybdenum than in the Mo_2_TiC_2_Al phase. Also, a small part of the Al is present in the form of the Al_4_C_3_ carbide phase. With high Al content (*x* = 10), the content of MAX phases decreases while the amount of conventional carbides (TiC) increases (Fig. S1A,† samples Mo12, Mo12Na).

Similar to the case of Mo- and Ti-based MAX phases/MXenes samples discussed in Section 3.1, porous composites were formed with a monolithic structure, suitable mechanical properties and the thickness of the etched layer reaching the thickness of the sample (3.5 mm) for the nominal ratio Ni : Mo : Ti : C : Al = 1 : 2 : 1 : 2 : 7 (sample Mo6), after etching in 10 M NaOH at room temperature during 60 days (Fig. 2E). The peaks in the diffraction patterns of the sample Mo6 (Fig. 2C) related to Al_5_Mo_2_Ni, Ni_2_Al_3_, Al_4_C_3_ disappear and related to Mo_2_TiAl_*x*_C_2_ MAX phase decrease after the etching of such composites in 10 M NaOH, and the appearance of new peaks is observed at 2*θ* ≈ 5° and 7.5° attributed to MXene, and at 2*θ* ≈ 44° related to nano-Ni (Fig. 2E, sample Mo6Na). Also, heating can facilitate the etching process and similar structure can be formed by the etching during 15 days at 70 °C (sample Mo6aNaT70, Table 1). After partial etching of the sample Mo6 in HF solution for 3 days, the appearance of two small-angle diffraction peaks is observed at 2*θ* ≈ 5° and 6° attributed to MXene (Fig. 2E, sample Mo6HF1). In literature, small-angle diffraction peak for non-delaminated Mo_2_TiC_2_T_*x*_ (T = OH, F) MXene is observed at 2*θ* ≈ 7° and for delaminated at 2*θ* ≈ 4-5°.^26,56^ Therefore, it can be assumed that the first peak at 2*θ* ≈ 5° (Fig. 2E, sample Mo6HF1) can be attributed to MXene obtained from modified MAX phase Mo_2_TiAl_*x*_C_2_ (probably Mo_2_TiAl_2_C_2_), and the second peak at 2*θ* ≈ 6° – to MXene obtained from conventional Mo_2_TiAlC_2_ MAX phase with one Al layer. After complete Al etching from the sample Mo6 in HF solution for 6 days the distance between the Mo_2_TiC_2_T_*x*_ (T = OH, F) layers decreases and one small-angle diffraction peak at 2*θ* ≈ 6° is observed (Fig. 2E, sample Mo6HF2). Therefore, taking into account the thickness of one Mo_2_TiC_2_T_*x*_ (T = OH, F) MXene layer approximately 1 nm^26,56^ and *d*-spacing at 2*θ* = 5° equal 1.7 nm, the average interlayer spacing between the MXene sheets should be equals to 0.7 nm. Therefore, an increase *d*-spacing in modified Mo_2_TiAl_*x*_C_2_ MAX phase facilitate of Al etching and formation of Mo_2_TiC_2_(OH)_*x*_ MXene in 10 M NaOH.

The results shown in Fig. 2B and C suggest that the presence of Ni in the reaction mixture can promote the formation of conventional and modified MAX phases (Mo_2_Ti_2_AlC_3_, Mo_2_TiAlC_2_, Mo_2_TiAl_*x*_C_2_). As an example, the content of MAX phases is higher and that of TiC is lower in the sample Mo6 with Ni compared to the sample Mo5 without Ni. The possible mechanism can include the formation of Al-rich metal–Al alloys with a lower melting point *e.g.* Ni_2_Al_3_ (*T*_melt_ = 1133 °C)^59^ that accelerates the diffusion of components and, therefore, formation of final products (MAX phases) and reduced content of intermediate compounds (TiC).

The particle size of the aluminium also significantly impacts the reaction products. When using micro-sized aluminium powder, the content of Mo–Ni–Al alloys in the reaction products tends to increase, while the presence of the Mo_2_TiC_2_Al MAX phase decreases (Fig. S1B,† sample Mo6Al_micro_). This may be because micro-sized Al powder begins reacting with Ni, Mo and Ti particles at lower temperatures, but only reacts with C particles at higher temperatures. However, nano-sized Al powder reacts at a higher temperature with all elements; the reaction could also be affected by the presence of the protective Al_2_O_3_ film on the surface of the Al nanoparticles (Fig. S1C†).^60^ After the etching process 15 days at 70 °C such sample mainly consists of Mo_2_Ti_2_AlC_3_ MAX phase, MXene and nano-Ni (Fig. S1B,† sample Mo6Al_micro_NaT70).

### 3.3 Electrodes structure and surface morphology

Table 2 compares some representative parameters of the samples Mo5, Mo6 after sintering and etching. Samples with and without Ni after formation and pressing (Fig. 1A) have the same diameter (25 mm) and approximately the same thickness (∼3.5 mm). After sintering, the sample without nickel shows volume expansion (Mo5, Fig. 1B, Table 2), lower density, and higher weight loss, which probably is caused by the evaporation of Al in all volume of the sample during the sintering process and the formation of the porous structure.^61^ Furthermore, during sintering, Al reacts with Mo, forming a Mo_3_Al_8_ alloy with a higher melting point (*T*_melt_ = 1570 °C) than the processing temperature (1500 °C), slowing down the formation of the MAX phase.

The sample containing nickel (Mo6, Fig. 1C, Table 2) after sintering undergoes slight shrinkage (reduction in diameter and thickness), probably due to the formation of Ni–Al alloys (Ni_2_Al_3_ with *T*_melt_ = 1133 °C and Al_5_Mo_2_Ni detected in sintered products), with a higher melting point than pure Al (*T*_melt_ = 660 °C), but lower than for Mo_3_Al_8_ (*T*_melt_ = 1570 °C) and TiAl_3_ (*T*_melt_ = 1340 °C). Therefore, the Ni_2_Al_3_ phase remains molten during the entire synthesis process (3 hours at 1500 °C) and plays the role of a sintering additive which promotes the interdiffusion of the components and accelerates the formation of final products (MAX phases). This effect is even more pronounced than in the case of MAX phase synthesis in the presence of Al excess.

On the other hand, the vapour pressure of Al in such Ni–Al alloy is lower^62–64^ which hinders the evaporation of Al and inhibits the process of pore formation. Thus, the addition of Ni to the initial reaction mixture affects not only the composition of the final sintered products but also the porous structure.

### 3.4 Structural, chemical and microstructural characterisation

**Fig. 3:**
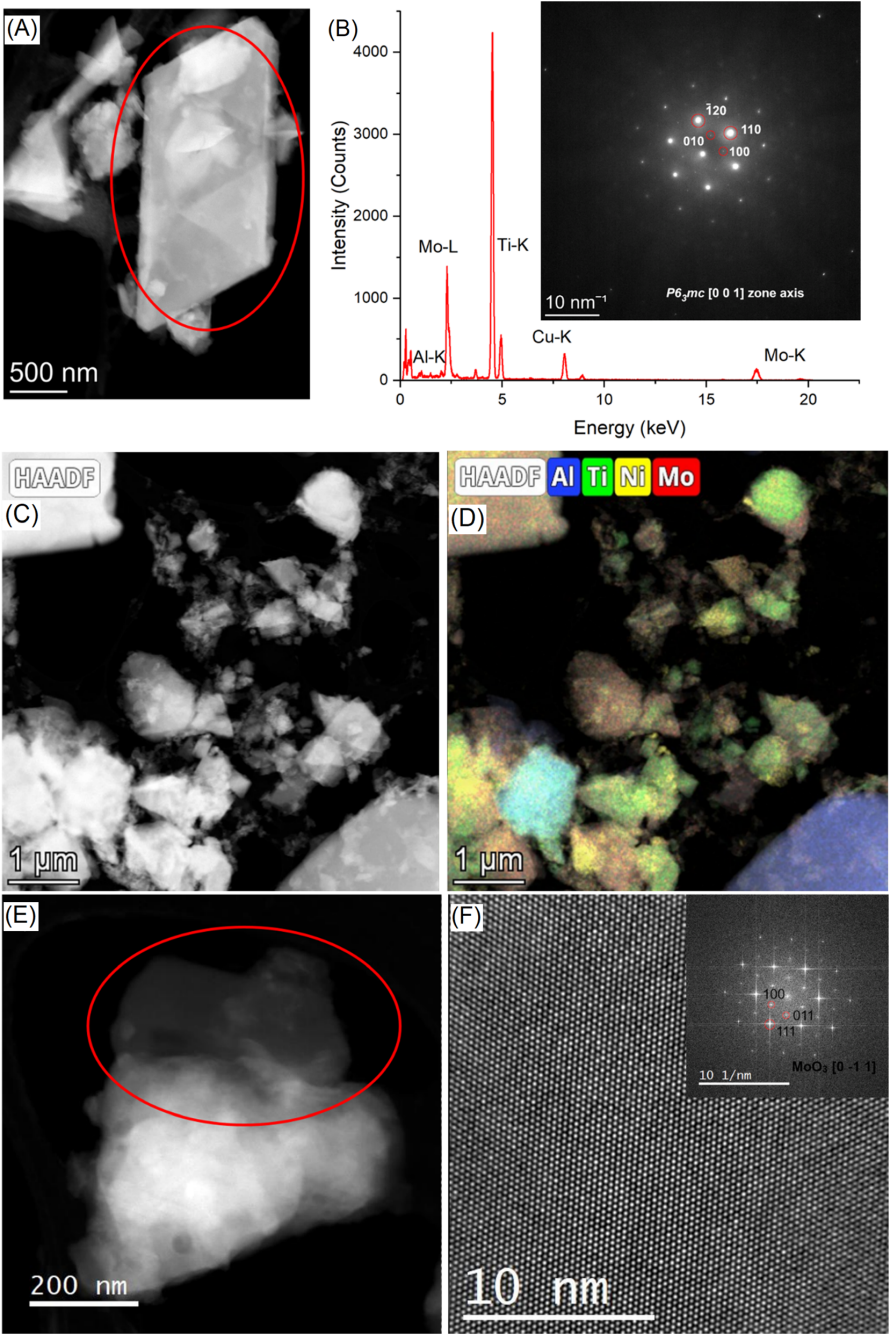
STEM-HAADF micrograph of the sample Mo5Na (A), the red circles highlight the area used to extract the corresponding EDS spectrum and SAED pattern (inset) (B). STEM-HAADF micrograph of the sample Mo6Na (C), and corresponding EDS chemical maps (D), STEM-HAADF micrograph of the sample Mo6Na (E), the red circle highlights the area used to acquire the HR-TEM image (F), the inset shows the corresponding FFT which has been successfully indexed by using the crystallographic structure of α-MoO_3_.

Fig. 3A shows the STEM-HAADF micrograph acquired on a flake of the sample Mo5Na; the corresponding EDS spectrum and SAED pattern (inset) are presented in Fig. 3B. EDS analysis confirms the presence of the crystals rich in Ti and Mo in the sample Mo5Na. It should be noted that for this crystal the Al content is below 0.7 at%. The corresponding SAED pattern (inset of Fig. 3B) has been successfully indexed by using the *P*6_3_*mc* structure seen along the [0 0 1] zone axis.^65^ It confirms the successful Al etching of the MAX phase and the formation of the MXene phase. Since the XRD data for the sample Mo5Na (Fig. 2B) suggests that the content of the Mo_2_TiAlC_2_ phase decreases after etching, the formed MXene can be described as Mo_2_TiC_2_(OH)_*x*_, also formation of Mo_2_Ti_2_C_3_(OH)_*x*_ is possible.

Fig. 3C and D shows the STEM-HAADF image and the corresponding EDS maps acquired on a flake of the sample Mo6Na. As expected, a strong heterogeneity of crystal sizes and compositions is revealed. In particular, many crystals present a low content of Al (typically below 3 at%). The presence of Ni-rich nanoparticles at the surface of the crystals can also be observed. The presence of thin nanocrystals of α-MoO_3_ (ref. 66) is also notable (Fig. 3E and F), in good agreement with the XRD analyses.

**Fig. 4:**
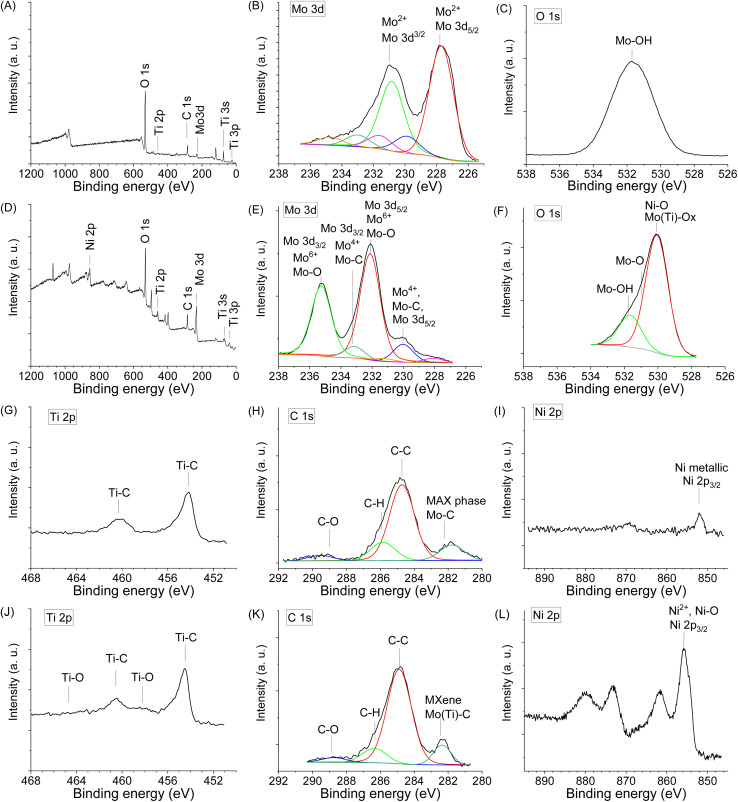
XPS spectra in different regions obtained for the sample Mo6 before etching (A, B, C, G, H and I), and Mo6Na after etching in 10 M NaOH (D, E, F, J, K and L).

X-Ray Photoelectron Spectroscopy (XPS) was used to study the Mo, Ti, Ni chemical states and the presence of surface functional groups (O, OH), which are crucial for electrochemical performance. The survey XPS spectra of the sample Mo6 before and Mo6Na after etching are shown in Fig. 4A and D, respectively.

Fig. 4B shows the high-resolution XPS spectrum of Mo 3d for the Mo6 sample before etching. There is a doublet (Mo 3d_5/2_–Mo 3d_3/2_) with binding energies of 227.7 and 230.8 eV related to Mo^2+^ components in Mo_2_TiAlC_2_ or Mo_2_Ti_2_AlC_3_ MAX phases.^50,67^ In this spectrum, other molybdenum species are observed. Mo^4+^ at 229.9 and 233.0 eV (Mo 3d_5/2_–Mo 3d_3/2_), and Mo^6+^ at 231.6 and 234.7 eV (Mo 3d_5/2_–Mo 3d_3/2_).

After partial Al etching (sample Mo6Na), the relative intensity of peaks related to MAX phases decreases, and new peaks near 230.0 eV (Mo^4+^, Mo 3d_5/2_) and 233.0 eV (Mo^4+^, Mo 3d_3/2_) related to Mo–C components in Mo_2_TiC_2_T_*x*_ (*x* = O, OH) MXene^14,68^ are observed (Fig. 4E). Other peaks can be assigned to molybdenum in oxidised phases, namely, the high relative intensity doublet at 232.3 and 235.4 eV related to Mo–O (Mo^6+^, Mo 3d_3/2_ and Mo 3d_3/2_), and a low intensity doublet Mo 3d_5/2_–Mo 3d_3/2_ at 227.8 and 230.9 eV, respectively and assigned to Mo^2+^.^67^

Thus, the combined studies show that molybdenum exists in sample Mo6Na in Mo–C species related to Mo_2_TiC_2_(OH)_*x*_ MXene obtained after etching of Mo_2_TiAlC_2_ MAX phase, and also in Mo^5+/6+^ (MoO_3_) oxidised form, most likely produced by the etching of Mo_3_Al_8_ phase and following oxidation. According to XRD analysis (Fig. 2C), the sample Mo6Na contains a considerable amount of conventional MAX phases. Therefore, the absence of peaks related to Mo_2_TiAlC_2_ and Mo_2_Ti_2_AlC_3_ MAX phases in the XPS spectra of sample Mo6Na might indicate that these phases are covered with MoO_3_ oxide and Mo_2_TiC_2_(OH)_*x*_ MXene phases.

The O 1s spectrum of sample Mo6 before etching (Fig. 4C) shows a peak corresponding to Mo–OH (531.5 eV). After etching, the sample Mo6Na (Fig. 4F) shows a very broad asymmetric peak, which is attributed to the variety of components related to oxygen-containing species such as Mo(Ti)–O (529.9 eV), Mo–O and Ni–O (531.0 eV), Mo–OH (532.0 eV).^14^

The Ti 2p core level spectrum obtained for the initial sample Mo6 (Fig. 4G) shows a peak at 454.4 eV related to Ti 2p_3/2_ components in Mo_2_TiAlC_2_ or Mo_2_Ti_2_AlC_3_ MAX phases.^50^ After etching, the position of this peak remains the same, and only a small additional peak at 457.8 eV related to Ti–O components appears, suggesting partial oxidation of titanium (Fig. 4I).

The C 1s signal for the sample Mo6 before (Fig. 4H) and after etching (Fig. 4K) shows the presence of carbon in different forms. The C 1s signal suggests a high C content in the initial sample. The presence of a peak which can be attributed to carbide arising from Mo_2_TiAlC_2_ or Mo_2_Ti_2_AlC_3_ MAX phases is detected at 282.5 eV. It can be assigned to C atoms bonded to outer Mo and inner Ti layers; an extra peak at 281.9 eV is possibly related to C in TiC.^50^ The other peaks correspond to C–C and CH_*x*_ species of unreacted C and contamination from the atmosphere; the presence of carbonate at 289.0 eV is also observed. The carbide peak arising from Mo_2_TiC_2_T_*x*_ (*x* = O, OH) MXene is detected at 282.6 eV (Fig. 4K). The contribution at 284.6 eV can be assigned to C–C, occurring probably due to the presence of unreacted carbon or carbon formed by Al_4_C_3_ etching in NaOH.^69^

A weak Ni 2p signal is observed in the spectrum of the initial sample before etching (Fig. 4I), with a main Ni 2p_3/2_ peak at 852.0 eV, a typical value for metallic Ni. The components related to Ni 2p_3/2_ (853.3 eV) corresponding to Ni_2_Al_3_ are also present in the spectrum. In the Ni 2p spectrum of the sample after etching (Fig. 4L), shows a main Ni 2p_3/2_ peak at 855.5 eV, a typical value for Ni^2+^ in Ni(OH)_2_.^70^ In general, the chemical environments derived from the XPS data corroborate the previous guidelines obtained from the XRD results.

**Fig. 5:**
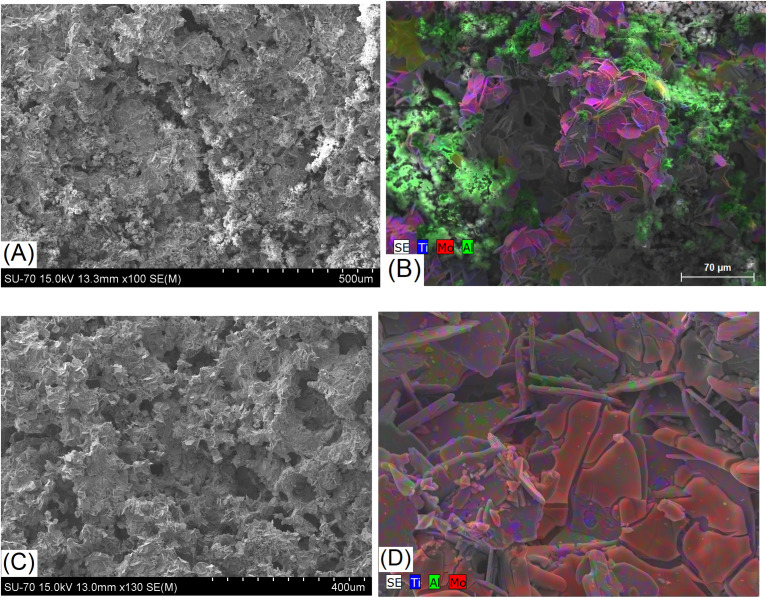
SEM/EDS images of the sample Mo5 after sintering and before etching (A and B), and sample Mo5Na after sintering and etching in 10 M NaOH (C and D).

**Fig. 6:**
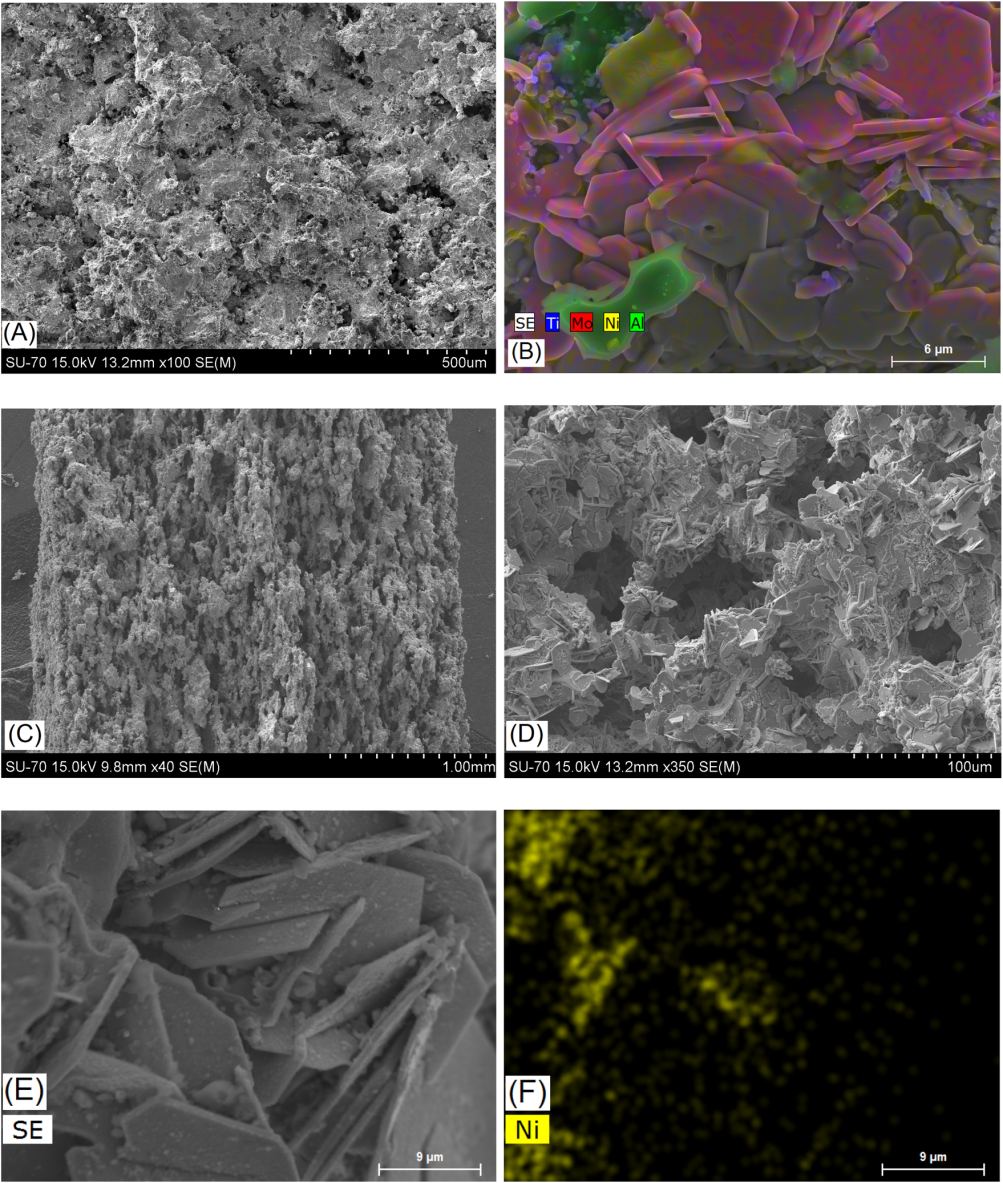
SEM/EDS images of the sample Mo6 with Ni after sintering, before etching (A and B), and sample Mo6Na with Ni after sintering and etching in 10 M NaOH (C–F).

The SEM studies coupled with EDS spectroscopy performed for the samples Mo5 (Fig. 5A and B) and Mo6 (Fig. 6A and B) after sintering show that some pores in the structure are already formed at the sintering stage of such composites. Aluminium evaporation takes place mainly from the surface of samples; therefore, the surface layer contains fewer layered phases and probably consists predominantly of conventional carbides. The thickness of such a layer is approximately 100 microns for the sample Mo6Na (Fig. S2†).

After Al etching and dealloying in 10 M NaOH, the samples Mo5Na (Fig. 5C and D) and Mo6Na (Fig. 6C and D) show more porous 3D monolithic structures consisting of multi-layered microcrystals. The macropores and mesopores are formed by the empty space between these chaotically distributed multi-layered crystals. A significant fraction of such macropores is open. Al etching and formation of MXene probably occurs along the perimeter of such multi-layered microcrystals, while the central part consists mainly of MAX phases and, within the proposed electrode concept, plays the role of catalyst support. The porous 3D structure of such electrodes is well-illustrated in Fig. 6C and D, confirming the formation of interconnected pores throughout the whole thickness of the electrode after etching.

SEM/EDS mapping of the sample Mo6Na shows the distribution of nanostructured Ni on the surface and inside the multi-layered MAX phase crystals after etching in 10 M NaOH (Fig. 6E and F).

SEM/EDS analysis of elemental composition in the sample Mo5Na before (Fig. S3, Table S1†) and after etching (Fig. S4, Table S2†) shows a considerable decrease in total Al concentration *C*(Al_before_)/*C*(Al_after_) = 8.24. At the same time, the Mo/Ti ratio does not undergo a significant change (Mo/Ti ratio in sample Mo5 = 1.3 and in Mo5Na = 1.6), which indicates that if the dissolution of molybdenum occurs, it should be minor. Therefore, taking into account the initial molar ratio in the sample Mo5 (Mo : Ti : C : Al = 2 : 1 : 2 : 7), the ratio of components in sample Mo5Na is close to Mo : Ti : C : Al = 2 : 1 : 2 : 0.85.

The reduction in total Al concentration in the sample Mo6Na after etching is about *C*(Al_before_)/*C*(Al_after_) = 3, not as high as might be expected, probably due to incomplete etching of Al from the MAX phase and Ni–Mo–Al alloy (SEM/EDS analysis data shown in Fig. S5, Table S3 and Fig. S6, Table S4†). The Mo/Ti ratio is around 1.8 for the sample Mo6 (after sintering) and 1.2 for Mo6Na (after etching). This suggests that a partial dissolution of the MoO_3_ phase might occur (the MoO_3_ peaks were not detected in the diffractogram of the sample Mo6Na, Fig. 2C).

### 3.5 Electrochemical characterisation and hydrogen evolution tests

**Fig. 7:**
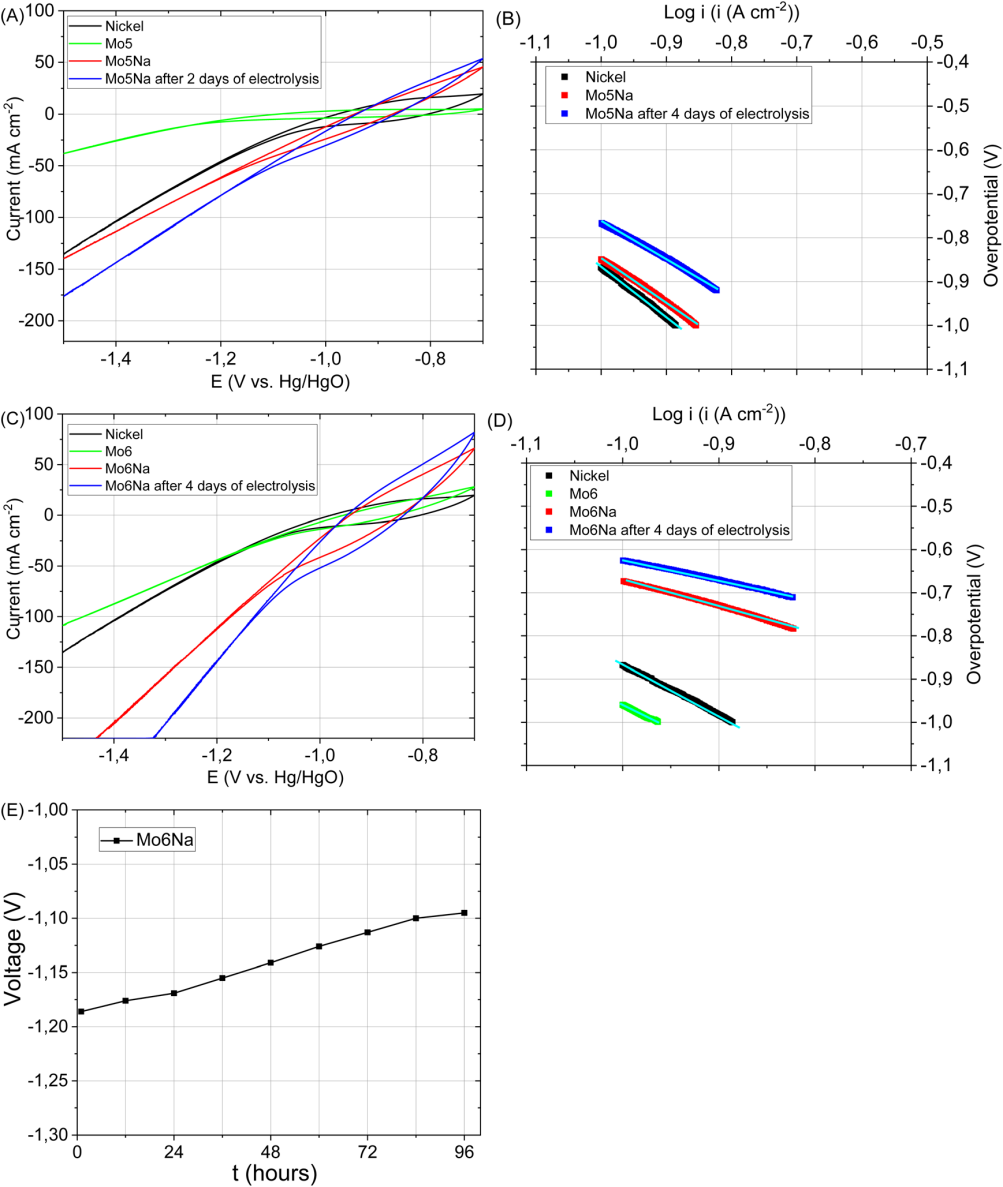
HER polarization curves recorded (at scan rate 10 mV s^−1^) for the following samples: pure metallic nickel, Mo5, Mo5Na, and Mo5Na after 4 days of electrolysis (A). Tafel plots for the HER (A) occurring on the electrodes (B), HER polarization curves recorded (at scan rate 10 mV s^−1^) for the following samples: nickel, Mo6, Mo6Na, and Mo6Na after 4 days electrolysis (C). Tafel plots for the HER (C) occurring on the electrodes (D), stability test of sample Mo6Na at constant current 1 A (E).

The catalytic activity of the electrode Mo5 (Fig. 7A and B) consisting predominantly of Mo_2_TiAlC_2_, Mo_2_Ti_2_AlC_3_ MAX phases, Mo_3_Al_8_, Al_4_C_3_ and TiC phases is notably higher than that of the electrode based on the Ti_3_AlC_2_ MAX phase.^37^ This is consistent with the literature data^12,71^ where the Mo_2_TiAlC_2_ MAX phase was highlighted as the most active HER catalyst among other MAX phases. According to,^58^ the Mo electrode stability decreases under neutral and basic conditions due to the formation of soluble surface species, but the dissolution rate is very low at −0.6 V *vs.* Ag/AgCl. Therefore, CV curves were recorded in the range of −0.7 to −1.5 *vs.* Hg/HgO to avoid considerable Mo ions oxidation and dissolution.

An even better electrocatalytic activity was observed for the sample Mo6 (Fig. 7C and D). The initial Mo6 sample, as well as Mo5, consists of several phases with potentially high activity, including Mo_2_TiAlC_2_, Mo_2_Ti_2_AlC_3_ MAX and Mo_3_Al_8_ phases. However, the Mo6 sample also includes Ni-containing phases (Al_5_Mo_2_Ni, Ni_2_Al_3_), which are likely responsible for the higher electrocatalytic activity.^72–74^ It should be noticed that the attained currents almost reach those observed for the Ni electrode, used as a reference for the target performance.

**Fig. 8:**
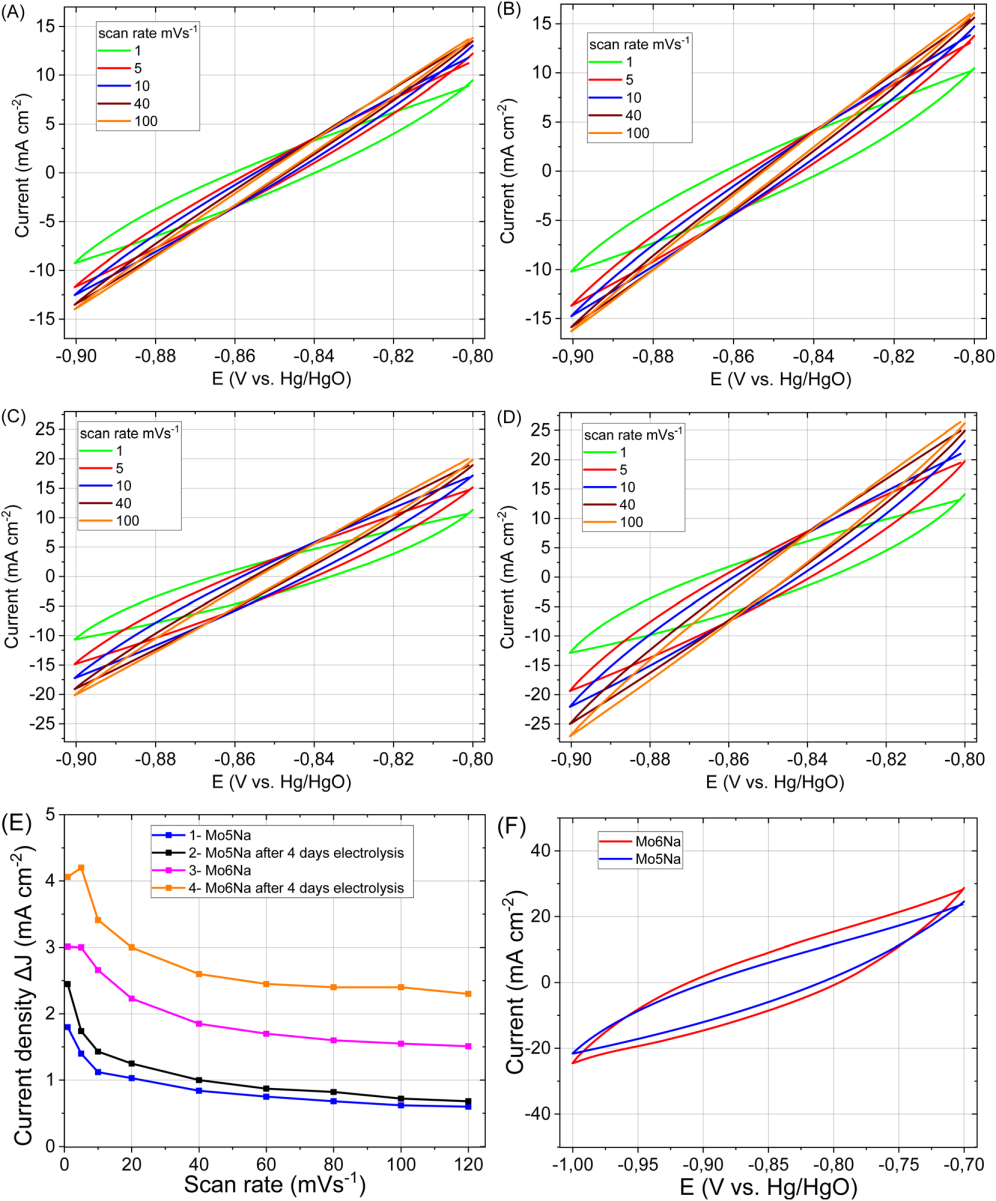
CV curves of the electrodes: Mo5Na (A), Mo5Na after 4 days electrolysis (B), Mo6Na (C), Mo6Na after 4 days electrolysis (D) at different scan rates. The differences in current density plots against scan rate for electrodes Mo5Na and Mo5Na after 4 days electrolysis, Mo6Na and Mo6Na after 4 days electrolysis (E). CV curves of electrodes after 4 days electrolysis Mo5Na and Mo6Na at scan rate of 1 mV s^−1^ (F).

After aluminium etching, the electrodes Mo5Na and Mo6Na demonstrate superior electrocatalytic activity (Fig. 7A–D, Table 3). Taking into account the phase composition, the results of microstructural and chemical characterisation and cyclic voltammetry results (Fig. 8A–E), this improvement towards HER activity is provided by the combined effect of several factors. The presence of active MAX phases, MXene, Mo oxides is at least partially responsible for this increase. Second, the increase of specific surface area and intercrystallite porosity, facilitating the access of the electrolyte through the entire volume of the electrode, is believed to be one of the key factors affecting HER activity. The presence of molybdenum oxide (MoO_3_) might also contribute positively to HER activity by the formation of oxygen vacancies under highly-reducing cathodic conditions and the concomitant increase in electronic conductivity^58,75^ Oxygen vacancies combined with Mo^5+^ ions are regarded active sites for HER in MoO_3−*x*_ materials.^76^ These factors are believed to be the most decisive for the high electrocatalytic activity of the samples Mo5Na and Mo6Na, including further improvement of HER activity after 4 days of electrolysis testing (Fig. 7E, Table 3).

**Table 3:** Kinetic parameters of the HER for samples Mo5Na, Mo6Na after etching, and Mo5Na-el, Mo6Na-el after 4 days electrolysis, calculated from voltammograms, in the range 100–150 mA cm^−2^, recorded in 1 M NaOH solution at 293 K

Samples (electrodes)	Tafel slope, *b* (V dec^−1^)	The exchange current density, *i*_0_ (A cm^−2^)	Transfer coefficient, *α*
Mo5Na	−1,03	1.53 × 10^−2^	0.056
Mo5Na-el	−0.865	1.32 × 10^−2^	0.067
Ni electrode	−0.871	9.66 × 10^−3^	0.066
Mo6Na	−0.626	8.55 × 10^−3^	0.092
Mo6Na-el	−0.487	5.28 × 10^−3^	0.119

It should be noticed that, in the case of the sample Mo6Na, the etching also affects the Al_5_Mo_2_Ni, Ni_2_Al_3_ phases, leading to the leaching of aluminium with subsequent formation of RANEY^®^ Ni and Mo oxides.^72^

Taking into account the results of the XPS analysis of the Mo6Na sample (Fig. 4L), it can be concluded that at least some part of Ni is in the oxidised state. Similarly to the case of the Mo5Na sample, the electrocatalytic activity of Mo6Na increases after further cathodic reduction during electrolysis of MoO_3_ to MoO_3−*x*_ and Ni oxides to metallic Ni.^77,78^

In general, a notable increase in current density was observed after 4 days of HER testing for both electrodes Mo5Na and Mo6Na (Fig. 8A–E, Table 3), but ECSA calculations from the corresponding CV data is hindered by the considerable increase in current density at low scan rate, as opposed to the expected decrease. It can be speculated that this behaviour might be promoted by a large amount of nanopores formed by interlayer space of Mo_2_TiC_2_(OH)_*x*_ MXene. These pores become accessible for electrolyte ions (Na^+^) only at a low scan rate, resulting in an increase in the current density. The charge storage mechanism of Mo_2_TiC_2_(OH)_*x*_ MXene is similar to the lithiation and sodiation process of Mo oxides, and consists of several reactions that can increases capacity in aqueous^79,80^ or non-aqueous^14^ electrolytes.

The values of *C*_dl_ and ECSA, evaluated at different scan rates, are listed in Table 4. The values calculated for the high scan rates (100 mV s^−1^) are likely related to the MAX phase crystals area of the samples. The oxygen vacancies-rich MoO_3−*x*_ present in such composites can also promote faster charge storage kinetics.^80^ Values at low scan rates (1–5 mV s^−1^) also include the contribution of MXene, along with redox processes at low scan rates (MoO_3_, NiO reduction/oxidation, MXene sodiation).^19,29^

**Table 4:** Estimated values of capacitance (*C*) and ECSA from CV curves (Fig. 8B and D) at different scan rates for samples Mo5Na and Mo6Na, and capacitance (*C*_3_) estimated from CV curves (Fig. 8F) of electrodes Mo5Na and Mo6Na

Electrodes after 4 days electrolysis	*C* at 100 mV s^−1^ (F cm^−2^)	ECSA at 100 mV s^−1^ (cm^2^ cm^−2^)	*C* at 10 mV s^−1^ (F cm^−2^)	ECSA at 10 mV s^−1^ (cm^2^ cm^−2^)	*C* at 1 mV s^−1^ (F cm^−2^)	ECSA at 1 mV s^−1^ (cm^2^ cm^−2^)	*C*_3_ at 1 mV s^−1^ (F cm^−2^)
Mo5Na	0.0072	180	0.143	3575	2.45	61 200	6.0
Mo6Na	0.024	600	0.341	8525	4.0	101 500	9.1
	(F g^−1^)	(cm^2^ g^−1^)	(F g^−1^)	(cm^2^ g^−1^)	(F g^−1^)	(cm^2^ g^−1^)	(F g^−1^)
Mo5Na	0.035	880	0.69	17 400	11.9	298 000	28.7
Mo6Na	0.093	2330	1.32	33 200	15.6	394 000	35.4

As reported earlier, pillared Mo_2_TiC_2_ MXene has a surface area of 202 m^2^ g^−1^ (ref. 14) and a specific capacitance of 212 F g^−1^.^81^ To evaluate the potential of obtained materials in supercapacitors application, the CV curves, in wider range (from −0.7 to −1 V), were recorded at a scan rate 1 mV s^−1^ (Fig. 8F), and from −0.4 to −1.25 V (Fig. S7†). The calculated values of capacitance (*C*_3_) are presented in Table 4. Thus, the proposed concept of the electrode design and processing allows obtaining high values of areal capacitance without using many additional steps or additives. Therefore, the obtained composites also represent a promising electrode material for energy storage systems such as metal (Li, Na)-ion batteries and supercapacitors.

**Fig. 9:**
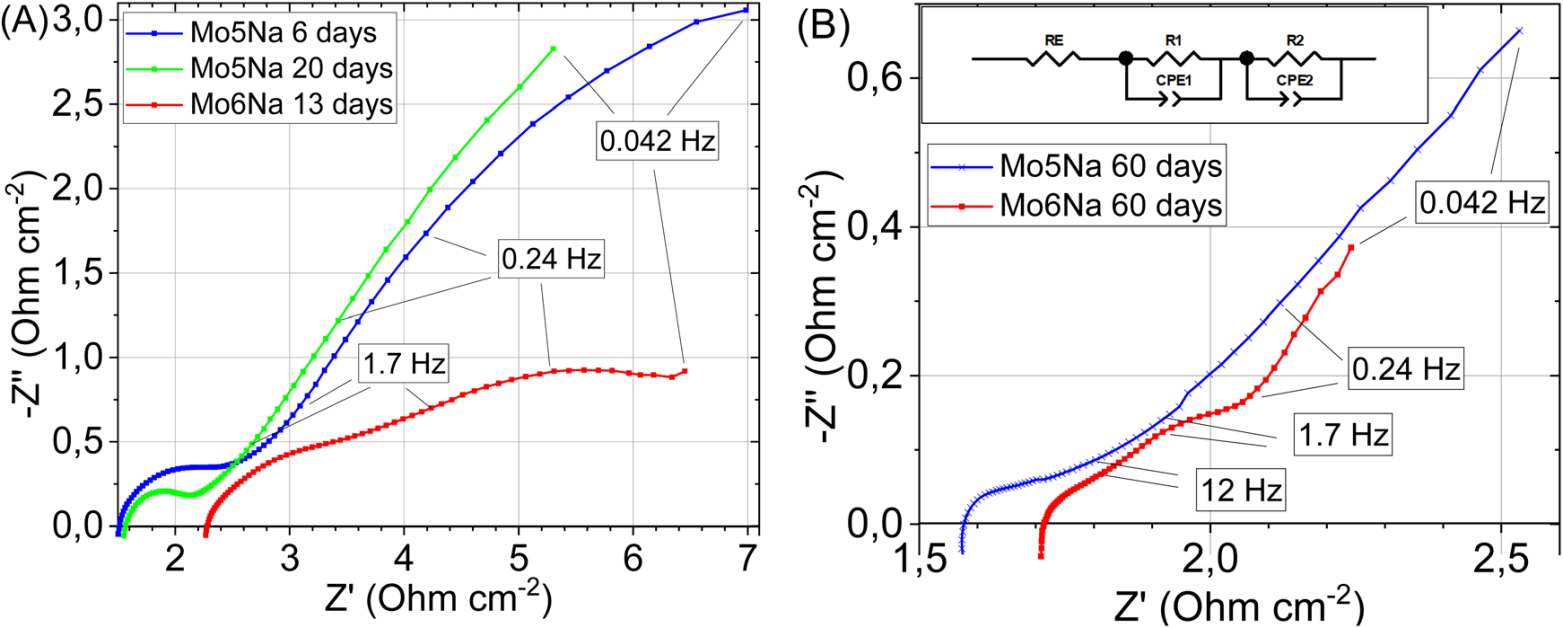
Electrochemical impedance spectroscopy data for the electrodes etched during different time: Mo5Na after 6 days and 20 days etching, and for sample Mo6Na after 13 days etching (A). Electrochemical impedance spectroscopy data and used equivalent circuit for samples Mo5Na, Mo6Na after 60 days etching (B).

To evaluate electrical conductivity and capacitance for the Mo5Na and Mo6Na electrodes, impedance spectra were recorded. The Nyquist plots showed that the electrical impedance of the samples decreases during etching (Fig. 9A and B), due to increased capacitance, and the sample Mo6Na containing Ni has a lower electrical impedance than the sample Mo5Na (without Ni).

After 60 days of etching (Fig. 9B), only one resolved semicircle can be observed in high-frequency region for the Mo5Na electrode and two semicircles for the Mo6Na electrode at the high and low frequency regions. These data were fitted using the represented equivalent circuit; the corresponding results are presented in Table 5. The sample Mo6Na, containing Ni, offers considerably higher areal capacitance (up to 0.95 F cm^−2^), which stems from the significantly higher electrical conductivity of the sample compared to the nickel-free sample Mo5Na. In general, it can be seen that better catalytic activity of the sample Mo6Na is provided by the combined effect of several factors, of which electrical conductivity appears to be the one of the most important.

**Table 5:** Metrics of the elements in equivalent circuit as a result of fitting EIS spectra

Electrodes	*R*_E_ (Ω cm^2^)	*R*_1_ (Ω cm^2^)	*C*_1_ (*n* = 1) (F cm^−2^)	*n*	*R*_2_ (Ω cm^2^)	*C*_2_ (*n* = 1) (F cm^−2^)	*n*
Mo5Na	3.1	0.26	0.005	0.53			
Mo6Na	1.18	0.15	0.040	0.61	0.146	0.95	0.87

Finally, it should be noticed that the prepared 3D porous electrodes, although they show a superior activity, do not show a gradual increase in overpotential over time, typical for the pure Ni electrodes under cathodic polarization in an alkaline medium and taking place due to the formation of nickel hydride layer on the active electrode surface.^7,82^

The latter indicates that the main boosting effect is provided by the components other than Ni in their composition, including the phases MAX, MXene and MoO_*x*_. It is believed that there is still a large space for tuning the 3D microstructural features and the arrangement of the MXene layers in such electrodes to further enhancement of the electrocatalytic effect towards water splitting.

## 4. Conclusions

In the present work, a simple preparation method of MAX phase/MXene/metal (Mo_2_Ti_2_C_3_Al/Mo_2_TiC_2_(OH)_*x*_/Ni) composite electrodes with porous 3D structure was proposed.

The variation of the carbon content with a high nominal Al excess (Mo : Ti : C : Al = 2 : 1 : *k* : 7, *k* = 2, 3, 4) allowed tuning Mo_2_TiC_2_Al and Mo_2_Ti_2_C_3_Al MAX phases ratio in sintered samples. It was found that the presence of Ni and Al excess in the reaction mixture promotes the formation process of conventional (Mo_2_Ti_2_AlC_3_, Mo_2_TiAlC_2_) and modified (probably Mo_2_TiAl_2_C_2_) MAX phases due to generating Al-rich metal–Al alloys (intermediates) with a lower melting point *e.g.* Ni_2_Al_3_, TiAl_3_, that accelerates the formation of final products (MAX phases) and reduce the content of intermediate compounds (TiC).

The presence of Ni in the initial reactional mixture allowed to reduce Al evaporation rate during the MAX phase synthesis and to increase the density of final products. The intentional excess of aluminium in the reaction mixture compared to the nominal composition of the MAX phases resulted in the formation of various metal–Al alloys (Mo_3_Al_8_, Ni_2_Al_3_, Al_5_Mo_2_Ni) segregated in the space between the MAX phase crystals. Al etching of such composites under alkaline conditions allowed to form composite electrodes with a high intercrystallite porosity. The possibility of Al etching from modified Mo_2_TiAl_*x*_C_2_ MAX phase in alkaline conditions, followed by the formation of MXene, was demonstrated; the associated etching times at ambient temperature corresponded to dozens of days. The obtained composite electrodes containing the MAX phases, MXenes and Ni showed a high electrochemical activity towards hydrogen evolution. Several factors contributed to this performance, including the intrinsically high electrocatalytic activity of the above phases, high specific surface area and intercrystallite porosity, promoted by compositional design facilitating the etching. The presence of nickel provided an additional important contribution, improving the electrical connection of the MAX phase and MXene crystals and the integrity of the obtained electrodes with a 3D structure.

## Conflicts of interest

There are no conflicts to declare.

## Supplementary Material

RA-014-D3RA07335A-s001
