# Supplementary material for: Composite MAX phase/MXene/Ni electrodes with a porous 3D structure for hydrogen evolution and energy storage application
Source: RSC Adv. 2024 Jan 18;14(5):3052–69. doi: 10.1039/d3ra07335a (PMC10795003; doi:10.1039/d3ra07335a)

Electronic Supplementary Material (ESI) for RSC Advances.
This journal is © The Royal Society of Chemistry 2024

Electronic Supplementary Information

Figure S1 – XRD of the samples: Mo12 after sintering (before etching), and Mo12Na after etching in 10M NaOH for 10 days (A); Mo6Al_{micro} after sintering (before etching), and Mo6Al_{micro}NaT70 after etching in 10M NaOH for 15 days at 70 °C (B); Al nano powder.

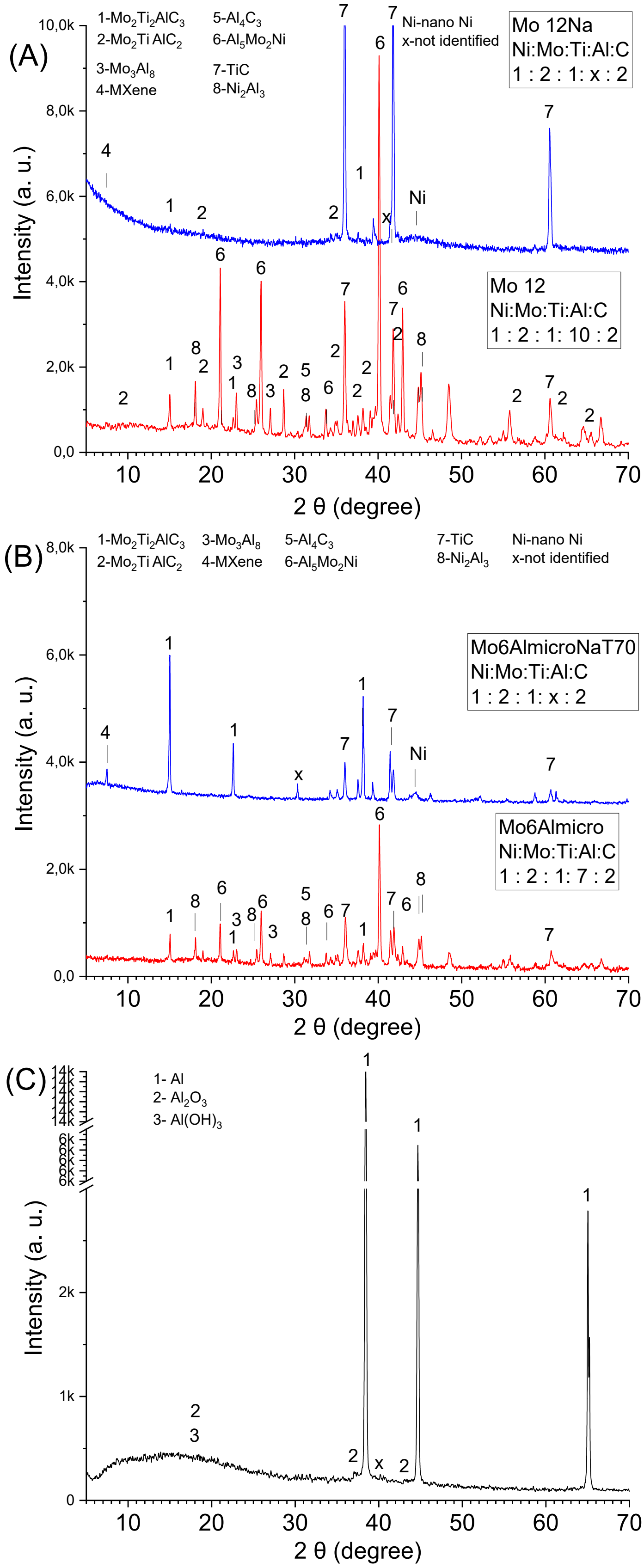

Figure S2 – SEM images of the surface layer of sample Mo6 after sintering (A, B, C, D).

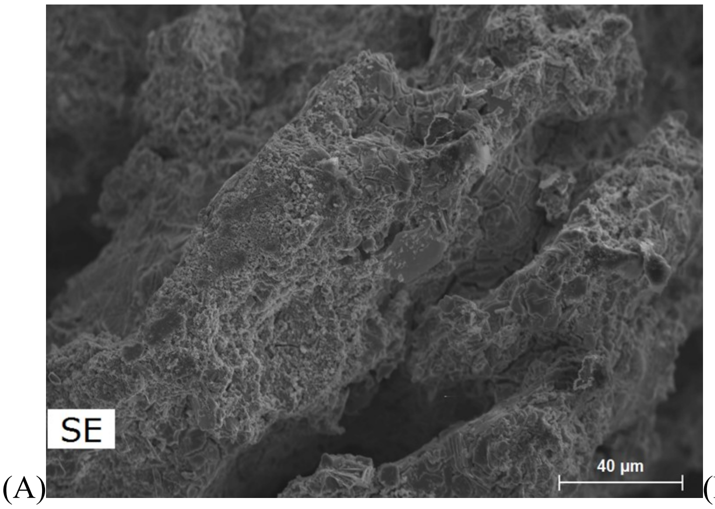

(A)

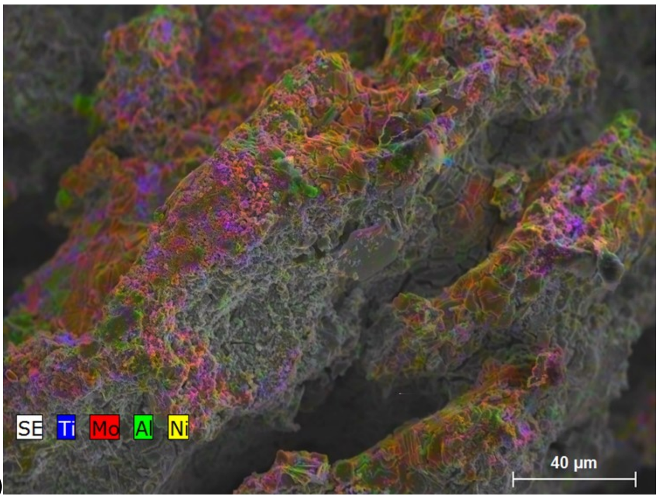

(B)

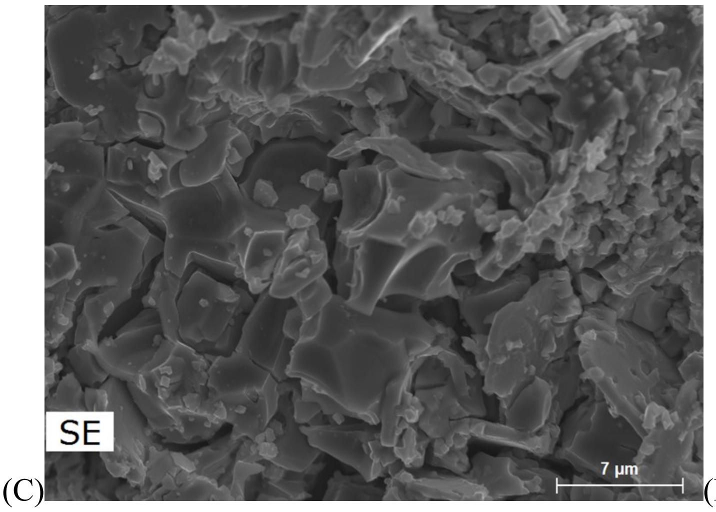

(C)

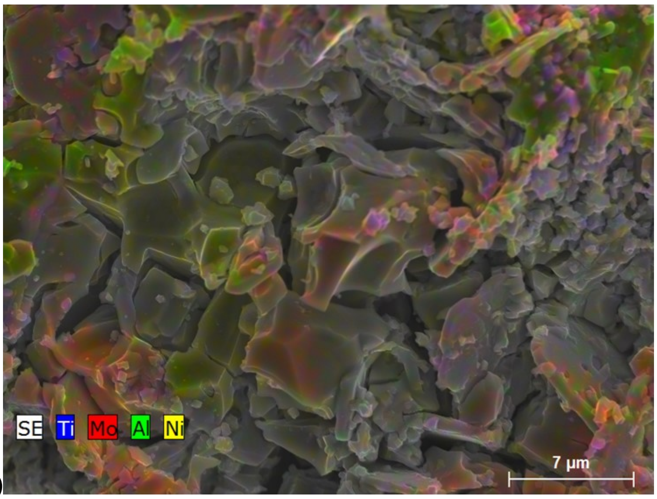

(D)

Figure S3 – EDS spectrum of sample Mo5 after sintering. The analysis was performed for the are shown by mapping image.

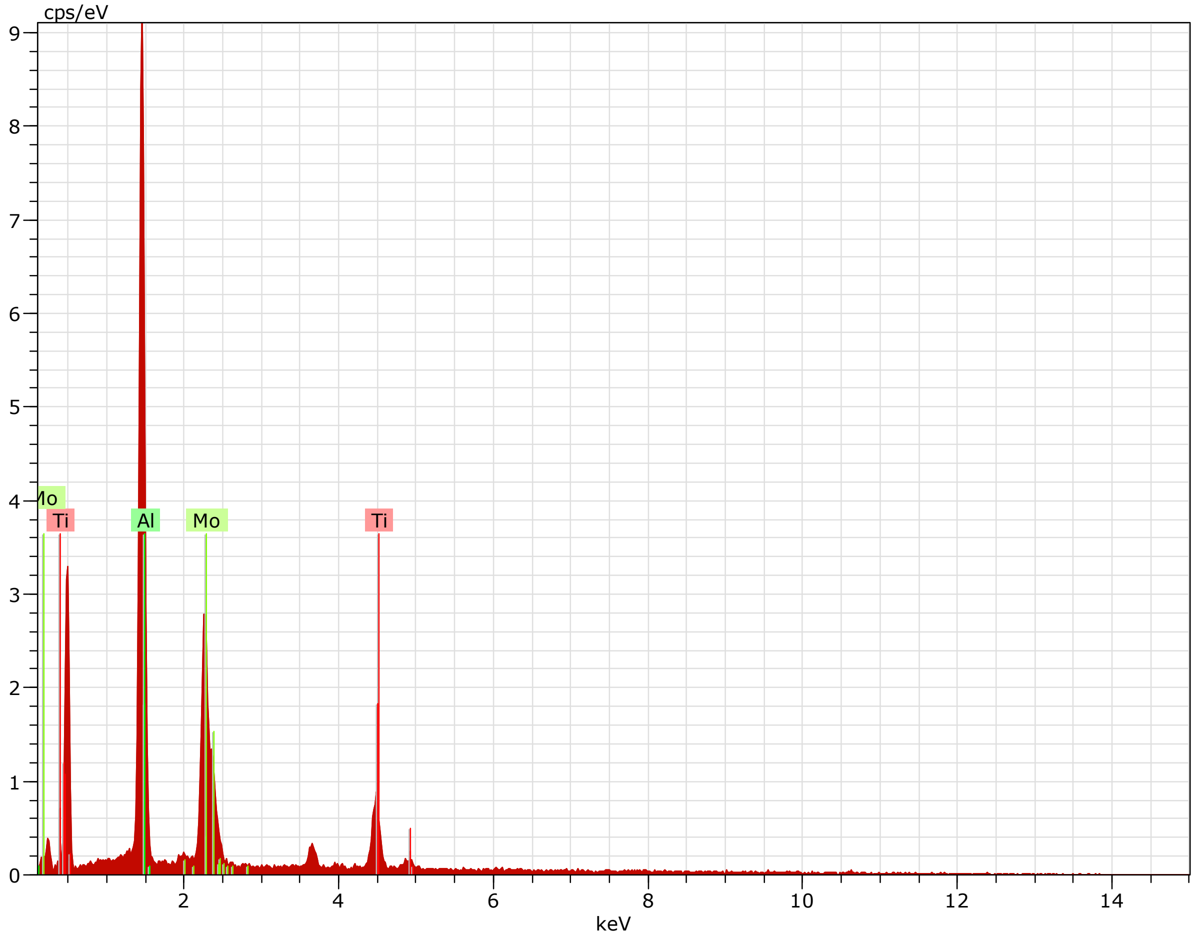

Table S1 – Quantitative results of the EDS analysis of sample Mo5 after sintering.

Element	Series	unn. [wt.%]	C norm. [wt.%]	C Atom. [at.%]	C Error (3 Sigma) [wt.%]
Titanium	K-series	9.40	16.46	15.08	1.10
Aluminium	K-series	22.85	40.02	65.04	3.33
Molybdenum	L-series	24.84	43.51	19.89	2.84

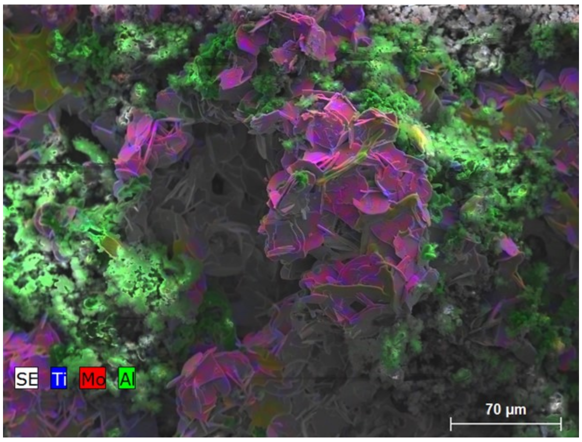

Figure S4 – EDS spectrum of sample Mo5Na after sintering and etching in 10M NaOH.

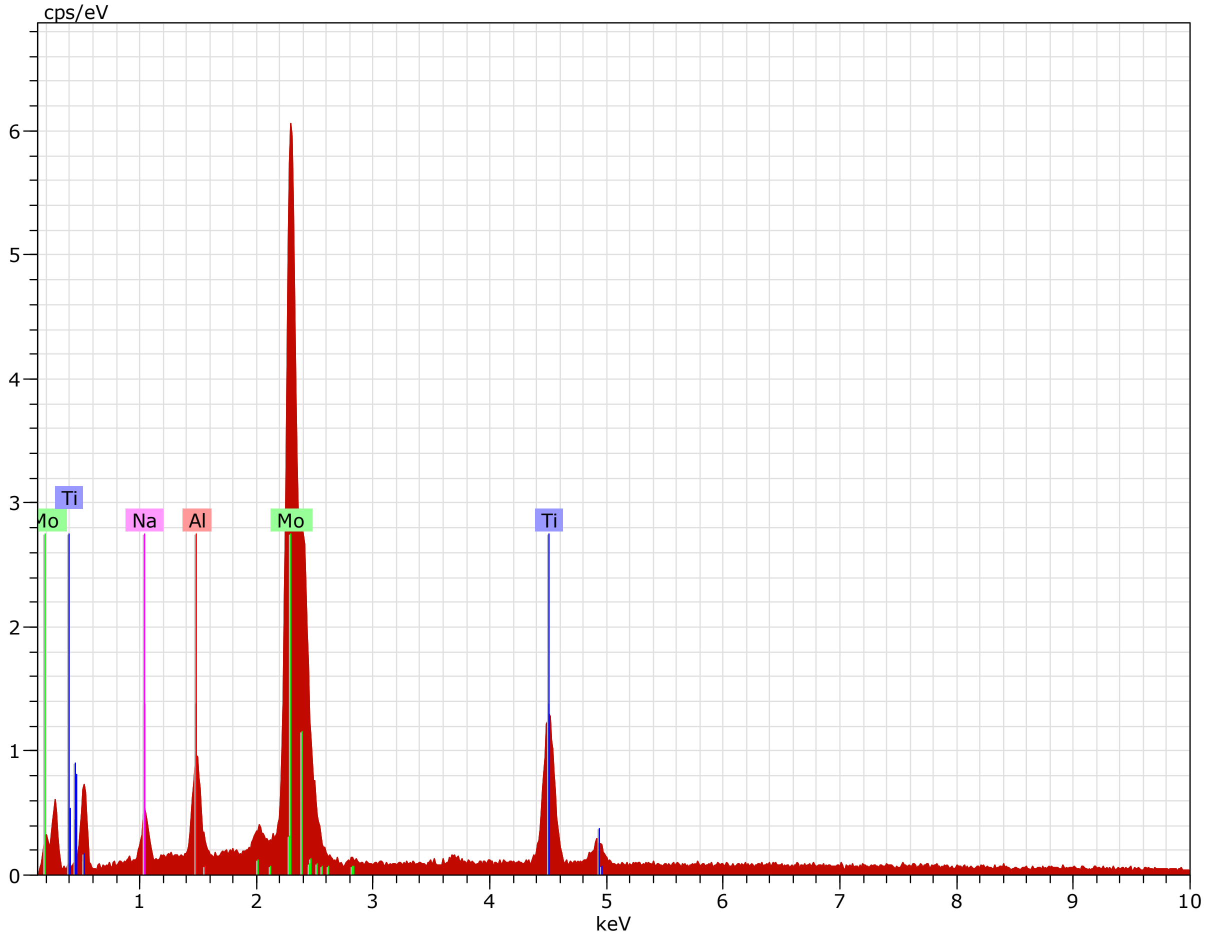

Table S2 – Quantitative results of EDS analysis of sample Mo5Na after sintering and etching in 10M NaOH. SEM image shows the analysed area.

Element	Series	unn. [wt.%]	C norm. [wt.%]	C Atom. [at.%]	C Error (3 Sigma) [wt.%]
Aluminium	K-series	2.10	3.02	7.89	0.41
Molybdenum	L-series	50.78	72.89	53.62	5.47
Titanium	K-series	15.48	22.22	32.76	1.52
Sodium	K-series	1.30	1.87	5.74	0.38

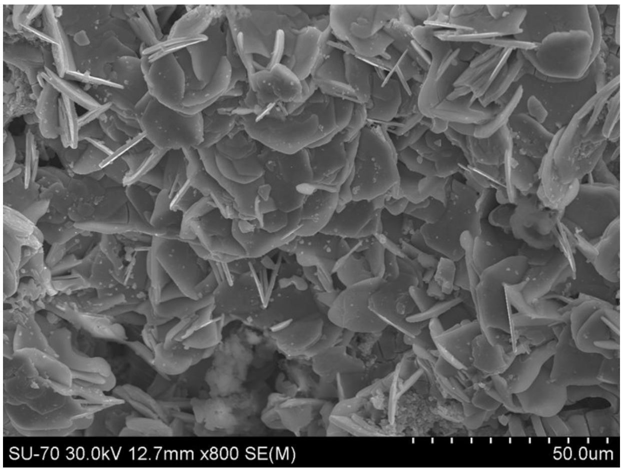

Figure S5 – EDS spectrum of sample Mo6 after sintering.

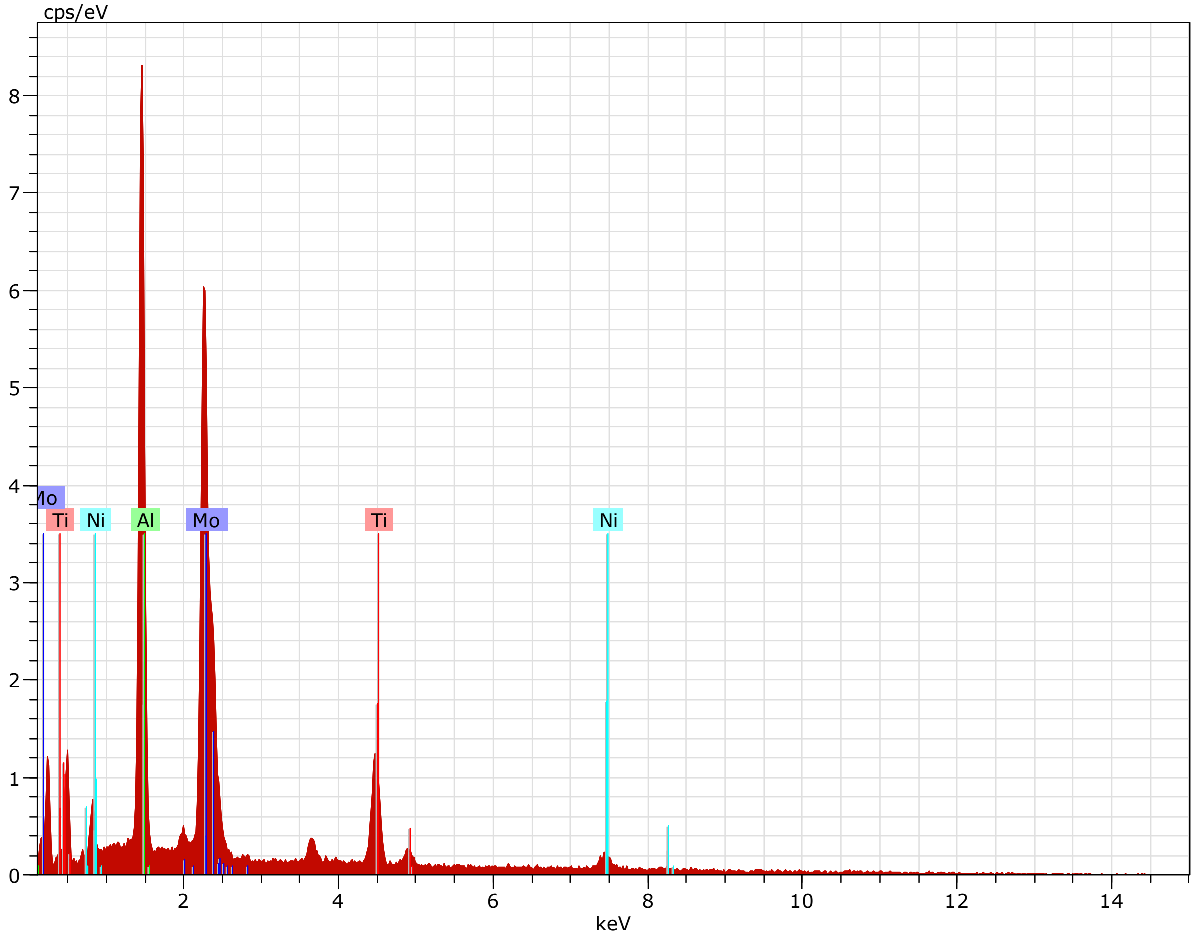

Table S3 – Quantitative results of EDS analysis of sample Mo6 after sintering.

Element	Series	unn. [wt.%]	C norm. [wt.%]	C Atom. [at.%]	C Error (3 Sigma) [wt.%]
Titanium	K-series	10.50	14.50	16.20	1.14
Aluminium	K-series	16.90	23.34	46.29	2.49
Molybdenum	L-series	39.23	54.18	30.22	4.30
Nickel	K-series	5.78	7.99	7.28	0.94

Figure S6 – EDS spectrum of sample Mo6Na after sintering and etching in 10M NaOH.

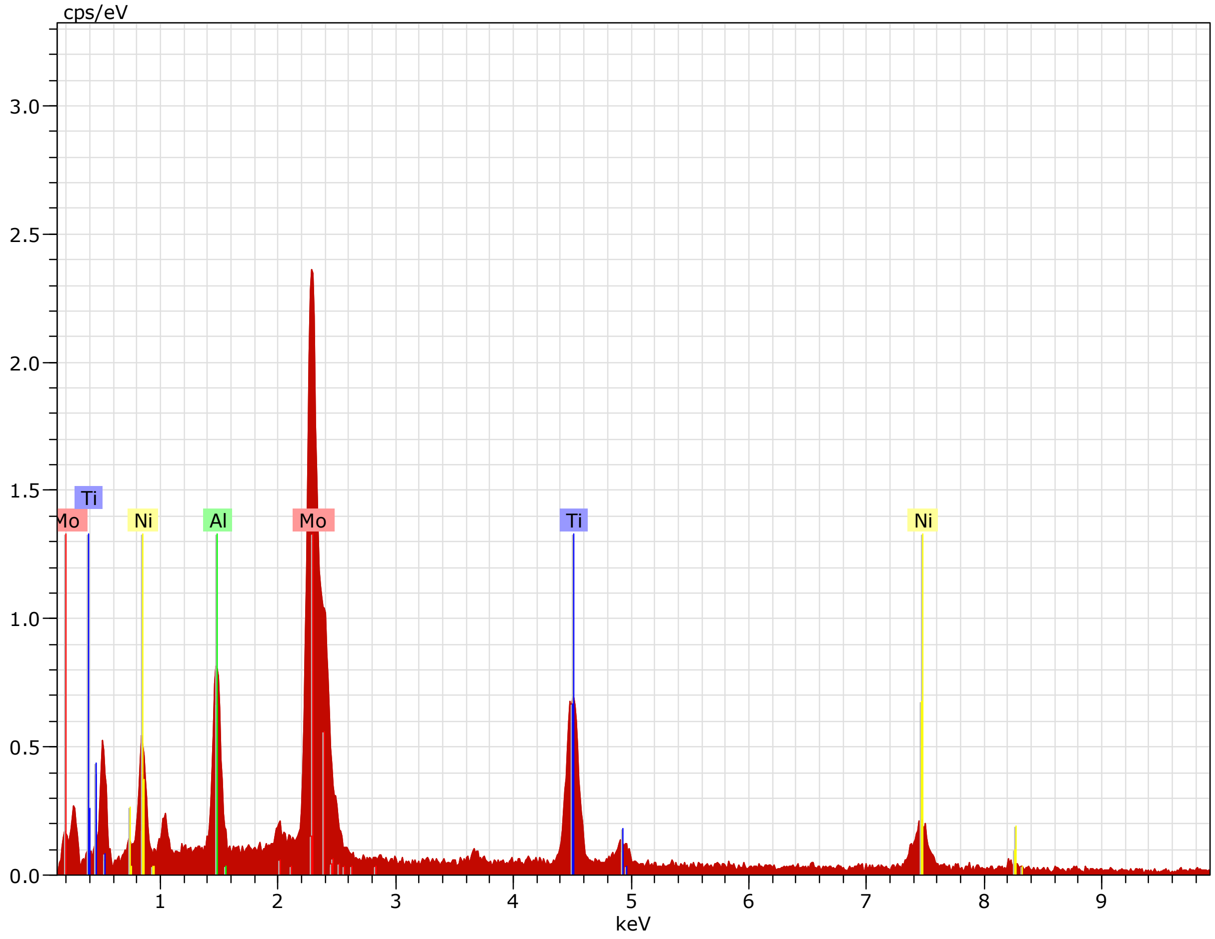

Table S4 – Quantitative results of EDS analysis of sample Mo6Na after sintering and etching in 10M NaOH.

El	AN	Series	unn. C [wt.%]	norm. C [wt.%]	Atom. C [at.%]	Error (1 Sigma) [wt.%]
Mo	42	L-series	44.95	50.83	33.56	1.65
Ti	22	K-series	18.70	21.14	27.97	0.64
Ni	28	K-series	19.06	21.55	23.26	0.79
Al	13	K-series	5.73	6.48	15.22	0.33

Figure S7 – CV curve of electrode Mo6Na after 4 days electrolysis at scan rate of 1 mVs^{-1}.

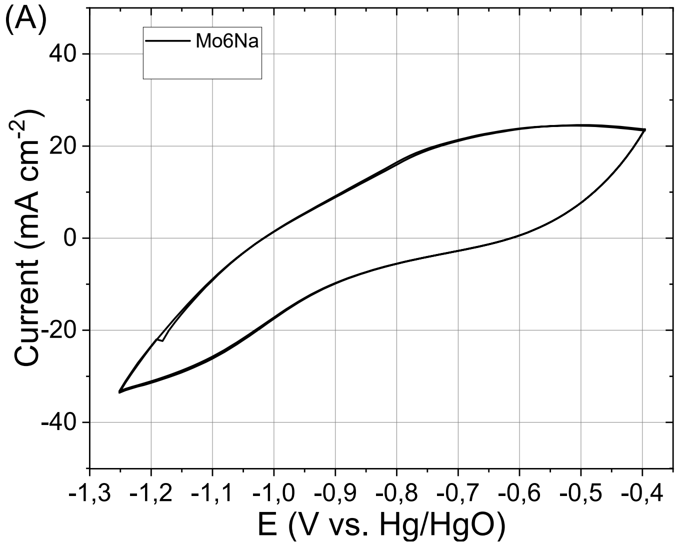

Supplement: RA-014-D3RA07335A-s001 [file RA-014-D3RA07335A-s001.pdf]
